# Supplementary material for: Bayesian nonparametric models characterize instantaneous strategies in a competitive dynamic game
Source: Nat Commun. 2019 Apr 18;10:1808. doi: 10.1038/s41467-019-09789-4 (PMC6472387; doi:10.1038/s41467-019-09789-4)
Supplement: Supplementary file 1 — Supplementary Information [file 41467_2019_9789_MOESM1_ESM.pdf]

Bayesian Nonparametric Models Characterize Instantaneous Strategies in a Competitive Dynamic Game

McDonald et al.

Supplementary Methods: Computer Opponent Algorithm

The computer opponent, also called the goalie, was represented by a vertical bar on the right hand side of the screen. The computer opponent used a fixed strategy based on a "track-then-guess" algorithm in which it initially attempts to match the vertical position of the puck at the start of each trial and subsequently chooses from a predefined set of strategies at random. The complexity of the resulting algorithm (Algorithm 2) is almost entirely due to two subtleties: (1) determining at what point the opponent should stop tracking and guess a strategy and (2) how this guess is chosen.

To address the first question, we consider the collection of points at which the puck has the potential to "outrun" the bar to one of the corners. For simplicity, our algorithm assumes that both players start from rest at the vertical middle of the screen and move maximally at each time step. After a certain position C_1 late in the trial, the distance the puck can move exceeds the bar's ability to catch it. After another point, C_2, near the end of the trial, the situation is reversed: the longer length of the bar allows it to block the puck more successfully. The two points C_1 and C_2 define each trial's *critical region*. Note, however, that because we have assumed both players start from rest at $y = 0$, this critical region is the same for each trial and calculated only once, at the start of the task. Algorithm 1 shows how this calculation is implemented.[1]

Algorithm 1: Calculation of critical and inflection points

Inputs: $T, \delta\theta, v_p$ `/* max time steps, velocity increment, puck speed */`

$\tilde{L} \leftarrow 10$ `/* typical lag; 75th percentile of distribution (Eq 1) */`
$\theta \leftarrow (1, 1, 1 + \delta\theta, \ldots, 1 + (T-2)\delta\theta)$ `/* cf. Equation (10), Equation (11) */`

$M_{puck} \leftarrow v_p \cdot (T, T-1, T-2, \ldots, 1)$ `/* maximum distance puck can move */`
$M_{bar} \leftarrow \frac{2}{3} v_p \cdot \mathsf{cumsum}(\theta) + \frac{\text{bar height}}{2}$ `/* maximum distance bar can move */`
$M_{bar} \leftarrow \mathsf{Lag}(M_{bar}, \tilde{L})$ `/* add opponent reaction time */`

$C_1 \leftarrow v_p \cdot \mathsf{FindFirst}(M_{puck} > M_{bar})$ `/* first point puck can outrace bar */`
$C_2 \leftarrow v_p \cdot \mathsf{FindLast}(M_{puck} > M_{bar})$ `/* last point puck can outrace bar */`

$(IP_1, IP_2) \leftarrow (C_1 - v_p, C_1 + 2v_p)$ `/* inflection points */`

return IP_1, IP_2, C_1, C_2

To determine when and what strategy the computer opponent chooses, our algorithm also defines a pair of *inflection points* surrounding the first critical point, C_1. The first of these, IP_1, is the horizontal position of the puck in the time step immediately before it reaches C_1, and the second, IP_2 is its location two time steps afterward. (Other choices are certainly possible; these were tuned to generate reasonable opponent play.) When the puck reaches IP_1, the computer chooses a two-part strategy (S, S') by sampling with replacement from a bag of strategies accumulated over the course of play. These two-part strategies allow the computer opponent to potentially counter strategies in which the participant reverses play, as well as adding unpredictability to the opponents' own movements.

[1] We have not included details related to the fact that neither avatar is allowed to move offscreen and that the bar's larger height give it an advantage in blocking at very late time steps. The latter is why the critical region does, in fact, end just prior to the end of the trial.

Having selected a strategy, the computer opponent then implements strategy S until the puck reaches IP_2, after which it implements S' until the end of the trial. After each trial, the bag of strategies is then updated by adding to it the participant's observed strategy on the most recent trial (see UpdateStrategies). In this way, the computer opponent biases its random strategy selection to adaptively counter the participant's observed tendencies.

Function UpdateStrategies

```
Strategies ← [(U, U), (D, D), Track ]
foreach trial do
    while trial not ended do
    |   play trial

    (S, S') ← { (U, U)    if puck was moving up at C1 and C2
                (D, D)    if puck was moving down at C1 and C2
                (U, D)    if puck was moving up at C1 and down at C2
                (D, U)    if puck was moving up down C1 and up at C2
                (Track)   else

    Strategies ← [Strategies, (S, S')]
```

These strategies differ only in where they set the opponent's desired bar position at the next time step. This can be thought of as the goal in a feedback control model with u_t as the control signal Equation (12) [1]. In the case of the tracking strategy, the goal is set at the participant's projected position in the next time step, while for the up and down strategies, u_t is maximal toward the top or bottom of the screen. Moreover, because the computer is capable of *perfectly* tracking the position of the puck, we implemented a variable lag in its behavior, comparable to a human's reaction time:

$$L = 0.12 + 0.1b \quad (1)$$

$$b \sim \text{Beta}(2, 5)\,. \quad (2)$$

That is, the lag, L is a random variable drawn separately for each trial. This variable as defined is measured in seconds, but is converted by the code to the integer indexing the closest time step. The resulting strategy execution is detailed in DoStrategy, while the full computer opponent algorithm is in Algorithm 2.

Function DoStrategy

```
Inputs: y_puck, L, y_ω          /* puck trajectory, lag (integer), bar location */
        S, v_ω                  /* strategy to execute, velocity next time step */

switch S do
    case Track do
        η ← 0
    case U do
        η ← 1
    case D do
        η ← −1

g ← y[t − L] + η · v_p                                      /* set goal */
u ← (g − y_ω) / v_ω                                         /* calculate control */
u ← sign(u) · min(1, |u|)                                   /* max control is 1 */

return u
```

Where: y_{puck}, L, y_ω; S, v_ω; $\eta \leftarrow 0$; $\eta \leftarrow 1$; $\eta \leftarrow -1$; $g \leftarrow y[t-L] + \eta \cdot v_p$; $u \leftarrow \frac{g - y_\omega}{v_\omega}$; $u \leftarrow \mathsf{sign}(u) \cdot \mathsf{min}(1, |u|)$.

Algorithm 2: Goalie algorithm

```
(S, S′) ← sample(Strategies)
for t = 1 to T do
    if x[t] < IP_1 then
        u ← DoStrategy(Track)

    if IP_1 < x[t] < IP_2 then
        u ← DoStrategy(S)

    if x[t] > IP_2 then
        u ← DoStrategy(S′)

MoveBar(u)                                   /* cf. Eq Equation (12) */
```

Supplementary Note 1: Normalization of Inputs to the Gaussian Process Model

In order to compare our sensitivity indices and model hyperparameters across model inputs, we normalized the units of our input variables to be $\mathcal{O}(1)$. More specifically, let the size of the screen be $H \times V$ pixels. Assuming H is larger (as it usually is), we define new, normalized screen coordinates $\tilde{x}_i$ by

$$\tilde{x}_i = \frac{2}{H}(x_i - \bar{x}_i) \tag{3}$$

with x_i the coordinate (either vertical or horizontal) and $\bar{x}_i$ the midpoint along the same dimension, both in pixels. This transformation rescales the game arena uniformly, maintaining the aspect ratio between horizontal and vertical dimensions.

Likewise, since the puck horizontal velocity is constant, and since the bar's vertical velocity is of the same order of magnitude, we measure all velocities in units of v_p. Thus the horizontal velocity of the puck is 1, and the vertical speed of the puck is ≤ 1.

Supplementary Note 2: Uncertainty of GP gradients

Here, we adopt the conventions of [2]: $\mathbf{y}$ is a vector of data points observed over a domain labeled by $\mathbf{x} \in \mathcal{X}$, $f \sim \mathcal{GP}(0, k(\mathbf{x}, \mathbf{x}'))$ is the latent Gaussian Process (GP), and $\mathbf{f}$ is the value of the GP at the data points. Let $\mathbf{X}$ denote the matrix of original data points and $\mathbf{K}$ the Gramian of these points ($K_{ij} \equiv k(\mathbf{x}_i, \mathbf{x}_j)$ for $\mathbf{x}_i, \mathbf{x}_j \in \mathcal{X}$). We would like to consider the value of $\mathbf{f}$ at points $\mathbf{X}_*$ distinct from the training set. From [2] (2.19), we have

$$\mathbf{f}_* | \mathbf{X}_*, \mathbf{X}, \mathbf{f} \sim \mathcal{N}(\mathbf{k}_*^\top \cdot \mathbf{K}^{-1} \cdot \mathbf{f}, \mathbf{k}_{**} - \mathbf{k}_*^\top \cdot \mathbf{K}^{-1} \cdot \mathbf{k}_*) \tag{4}$$

where we have additionally defined the matrix $\mathbf{k}_* \equiv k(\mathbf{X}_*, \mathbf{X})$ (this is a vector when $\mathbf{X}_* = x_*$, a single point) and $\mathbf{k}_{**} \equiv k(\mathbf{X}_*, \mathbf{X}_*)$.

Our goal is to calculate the distribution of the gradient of f, ∇f, with respect to $\mathbf{x}$:

$$\nabla_{\mathbf{h}} f = \lim_{\mathbf{h} \to \mathbf{0}} \frac{f(\mathbf{x} + \mathbf{h}) - f(\mathbf{x})}{\mathbf{h}} \tag{5}$$

where the division is elementwise division. We calculate this by considering $\mathbf{X}_* = [\mathbf{x} + \mathbf{h}, \mathbf{x}]$ (each point comprising one column), in which case the variable of interest, $\nabla_h f$ has mean $\lim_{h \to} \mathbf{A} \cdot \mathbf{f}_* / h$ with $\mathbf{A} = (1, -1)$. That is,

$$\mathbb{E}\left[\nabla_{\mathbf{h}} f\right] = \lim_{\mathbf{h} \to \mathbf{0}} \frac{\left(k(\mathbf{x} + \mathbf{h}, \mathbf{X}) - k(\mathbf{x}, \mathbf{X})\right)^\top \cdot \mathbf{K}^{-1} \cdot \mathbf{f}}{\mathbf{h}} = (\nabla_{\mathbf{h}} k)^\top \cdot \mathbf{K}^{-1} \cdot \mathbf{f} \tag{6}$$

That is, the calculation for the mean is the same as in the prediction case, with the kernel k replaced by its gradient. (Note once again that $f(\mathbf{x})$ is our Gaussian process, while $\mathbf{f}$ is the vector of observations of this GP at the training data points. Moreover, from here on, we will only consider evaluating the GP at a single text point $\mathbf{x}$.)

For the covariance matrix, we will make use of the fact that for a bivariate normal distribution

$$\text{var}(x_1 - x_2) = \sigma_1^2 + \sigma_2^2 - 2\sigma_{12} \tag{7}$$

with σ_i the standard deviation of each variable and σ_{12} the covariance of the two.

That is, the variance of the difference $f(\mathbf{x}+\mathbf{h}) - f(\mathbf{x})$ can be written

$$\Sigma_h = A - B \tag{8}$$
$$A = k(\mathbf{x}+\mathbf{h}, \mathbf{x}+\mathbf{h}) + k(\mathbf{x}, \mathbf{x}) - 2k(\mathbf{x}+\mathbf{h}, \mathbf{x}) \tag{9}$$
$$B = \mathbf{k}(\mathbf{x}+\mathbf{h})^\top \cdot \mathbf{K}^{-1} \cdot \mathbf{k}(\mathbf{x}+\mathbf{h}) + \mathbf{k}(\mathbf{x})^\top \cdot \mathbf{K}^{-1} \cdot \mathbf{k}(\mathbf{x}) - 2\mathbf{k}(\mathbf{x}+\mathbf{h})^\top \cdot \mathbf{K}^{-1} \cdot \mathbf{k}(\mathbf{x}) \tag{10}$$

where we have again used k as the kernel and $\mathbf{k}(\mathbf{x}) = k(\mathbf{x}, \mathbf{X})$, the vector formed by evaluating the kernel on the new datum and the original training set.

To calculate the covariance of (5), we then write $f(\mathbf{x}+\mathbf{h}) - f(\mathbf{x}) = \mathbf{h}^\top \mathbf{\Sigma}_{\nabla f} \mathbf{h}$ and take the $\mathbf{h} \to \mathbf{0}$ limit. It is straightforward to show that all terms of $\mathcal{O}(h)$ cancel, and

$$\mathbf{\Sigma}_{\nabla f} = \mathbf{A}_2 - \mathbf{B}_2 \tag{11}$$
$$\mathbf{A}_2 = \frac{\partial^2 k}{\partial \mathbf{x}^2} + \frac{\partial^2 k}{\partial \mathbf{x}'^2} + \frac{\partial^2 k}{\partial \mathbf{x} \partial \mathbf{x}'} - 2\frac{\partial^2 k}{\partial \mathbf{x}^2} = \frac{\partial^2 k}{\partial \mathbf{x} \partial \mathbf{x}'} \tag{12}$$
$$\mathbf{B}_2 = 2\mathbf{k}'' \cdot \mathbf{K}^{-1} \cdot \mathbf{k} + \mathbf{k}' \cdot \mathbf{K}^{-1} \cdot \mathbf{k}' - 2\mathbf{k}'' \cdot \mathbf{K}^{-1} \cdot \mathbf{k} = \mathbf{k}' \cdot \mathbf{K}^{-1} \cdot \mathbf{k}' \tag{13}$$

where we have again used the fact that k is symmetric in its arguments, so $\frac{\partial^2 k}{\partial \mathbf{x}^2} = \frac{\partial^2 k}{\partial \mathbf{x}'^2}$. Putting all this together, we then conclude that

$$\nabla f \sim \mathcal{N}((\nabla \mathbf{k})^\top \cdot \mathbf{K}^{-1} \cdot \mathbf{f},\ \nabla\nabla' k - \nabla \mathbf{k}^\top \cdot \mathbf{K}^{-1} \cdot \nabla \mathbf{k}) \tag{14}$$

Here again, we see that this is the same as the formula (4) for a new observation at $\mathbf{x}_*$ with the substitutions

$$k \to \nabla\nabla' k \tag{15}$$
$$\mathbf{k} \to \nabla \mathbf{k} \tag{16}$$

though the fact that ∇f is a *vector* means that $p(\nabla f | \mathbf{x}_*, \mathbf{f}, \mathbf{X})$ is *multivariate* Gaussian.

Supplementary Note 3: Precomputing kernels

For computational efficiency, it may be preferable to calculate kernel gradients explicitly, rather than relying on automatic differentiation. (For example, TensorFlow does not easily facilitate the computation of gradients of tensors, only scalars.) Here, we provide explicit formulas in the case that k is chosen to be the radial basis function (RBF) kernel:

$$k(\mathbf{x}, \mathbf{x}') = \sigma^2 \exp\left(-\frac{1}{2}\sum_{i=1}^{d} \frac{(x_i - x_i')^2}{\lambda_i^2}\right) \tag{17}$$

In which case we have

$$[\nabla\nabla' k]_{ij} = k(\mathbf{x}, \mathbf{x}') \left[\frac{\delta_{ij}}{\lambda_i^2} - \frac{(x_i - x_i')(x_j - x_j')}{\lambda_i \lambda_j}\right] \tag{18}$$

which implies

$$[\nabla\nabla' k]_{ij}\,|_{x'=x} = k(\mathbf{x}, \mathbf{x})\frac{\delta_{ij}}{\lambda_i^2} = \sigma^2 \frac{\delta_{ij}}{\lambda_i^2} \tag{19}$$

Similarly, we have

$$[\nabla \mathbf{k}]_{im} = [\nabla_x k(\mathbf{x}, x_m)]_i = -\frac{(x_i - (x_m)_i)}{\lambda_i^2} k(\mathbf{x}, x_m) = \boldsymbol{\lambda}^{-2} \odot (\mathbf{X} - \mathbf{x}) \odot \mathbf{k} \tag{20}$$

where we have used $\odot$ for the Hadamard (elementwise) product and assumed broadcasting in the i and m indices. Note also that these gradient formulas indicate that we naturally expect the scaling depicted in Figure 4B,C: gradient means and covariances are inversely proportional to the hyperparameters λ_i, so aggregated sensitivities should also roughly scale as powers of the hyperparameters.

Now, if we want to consider cases where prediction at more than one point is needed, we must be careful about indices. For example, the double gradient of the kernel evaluated at the new points is

$$[\nabla\nabla' k(\mathbf{x}, \mathbf{x}')]_{ij} \,|_{x=x_p, x'=x_{p'}} = k(x_p, x_{p'}) \left[\frac{\delta_{ij}}{\lambda_i^2} - \frac{(x_p - x_{p'})_i (x_p - x_{p'})_j}{\lambda_i, \lambda_j}\right] \tag{21}$$

with i, j labeling coordinates and p, p' new data points. Note again that in the special case that the new evaluation points are the *same* along the gradient directions, this reduces to

$$[\nabla\nabla' k(\mathbf{x}, \mathbf{x}')]_{ij} \,|_{x=x_p, x'=x_{p'}} = k(x_p, x_{p'}) \frac{\delta_{ij}}{\lambda_i^2} \tag{22}$$

Similarly,

$$[\nabla \mathbf{k}]_{pmi} = -\frac{(x_p - x_m)_i}{\lambda_i^2} k(x_p, x_m) \tag{23}$$

again with i labeling coordinates, p new data points, and m original data/inducing points. From this it follows that

$$\left[\nabla \mathbf{k}^\top \mathbf{K}^{-1} \nabla \mathbf{k}\right]_{ij,pp'} = \sum_n v_{pni} v_{p'nj} \tag{24}$$

$$v_{pni} \equiv \sum_m \frac{(x_p - x_m)_i}{\lambda_i^2} L^{-1}_{nm} k(x_m, x_p) \tag{25}$$

However, in inverting the covariance, we need a matrix, not a 4-tensor indexed by ij, pp'. So to accomplish this, we "stack" the datapoint and coordinate indices:

$$[\nabla\nabla' k]_{ij,pp'} \to k(X_p, X_p) \otimes \boldsymbol{\lambda}^{-2} \tag{26}$$

with $\otimes$ the Kronecker product and $\boldsymbol{\lambda}$ the diagonal matrix with entries λ_i. As for v, if one ensures that n is the *first* index, then one can simply `tf.reshape(v, [-1, D])` where `D` is the dimension of the vector x.

Supplementary Note 4: Uncertainties for sensitivity metrics

We have defined the sensitivity of the participant to the opponent's actions as $\varsigma = \|\nabla_g f\|^2$ where $f(\mathbf{x}) = \Phi^{-1}(\pi(\mathbf{x}))$ is the GP defining the local likelihood of a change point and ∇_g represents the gradient of f with respect to the opponent's state variables (position and velocity). If we let $\mathcal{X}_g$ denote the opponent's state variables, then by definition

$$\varsigma = \|\nabla_g f\|^2 = \nabla_g f \cdot \nabla_g f = \sum_{x \in \mathcal{X}_g} (\nabla_i f)^2 \tag{27}$$

That is, our sensitivity is a quadratic combination of gradients, $\nabla f^\top \cdot \mathbf{A} \cdot \nabla f$, with $\text{cov}[\nabla f] = \boldsymbol{\Sigma}_{\nabla f}$ as defined in (14). This combination has a generalized χ^2 distribution [3] with metric

$$(\mathbf{A})_{ij} = \begin{cases} \delta_{ij} & x_i, x_j \in \mathcal{X}_g \\ 0 & \text{otherwise} \end{cases} \tag{28}$$

and covariance $\boldsymbol{\Sigma}_{\nabla f}$.

Using $\varsigma = \|\nabla_g f\|^2$ as a measure of the sensitivity of the participant to the opponent is intuitive but also arbitrarily chooses an equal weighting of opponent position and velocity sensitivities. For a more principled solution, we adopted an alternative definition: $\varsigma' \equiv \|\nabla_g f\|_M^2$ with $\|\cdot\|_M$, with the Mahalonobis norm of $\nabla_g f$. That is,

$$\varsigma' = \|\nabla_g f\|_M^2 = \nabla_g f^\top \Sigma_g^{-1} \nabla_g f = \|\mathbf{L}_g^{-1} \nabla_g f\|^2 \tag{29}$$

with g subscripts once again restricting to variables in $\mathcal{X}_g$ and $\boldsymbol{\Sigma}_g = \mathbf{L}_g \mathbf{L}_g^\top$ (i.e., $\mathbf{L}_g$ is the Cholesky factor of $\boldsymbol{\Sigma}_g$). In what follows, we will drop the g subscripts for brevity.

We can motivate this choice by beginning with the observation (14) that $\nabla \mathbf{f}$ is multivariate normal with mean and covariance

$$\mu = \nabla \mathbf{k}^\top \mathbf{K}^{-1} \mathbf{f} \tag{30}$$

$$\Sigma = \nabla \nabla' k|_{x=x'} - \nabla \mathbf{k}^\top \mathbf{K}^{-1} \nabla \mathbf{k} \tag{31}$$

in which case $\equiv \mathbf{L}^{-1}(\nabla f - \boldsymbol{\mu})$ is multivariate normal with mean 0 and unit covariance matrix, or equivalently, $\mathbf{L}^{-1}\nabla f$ is multivariate normal with mean $\mathbf{L}^{-1}\mu$ and unit covariance. More importantly, what follows is that the sum of squares $\|L^{-1}\nabla f\|^2$ is a sum of squares of independent unit normals (with nonzero means) and thus follows a noncentral χ^2 distribution:

$$\|\mathbf{L}^{-1}\nabla f\|^2 = \|\nabla f\|_M^2 = \boldsymbol{\mu}^\top \boldsymbol{\Sigma}^{-1} \boldsymbol{\mu} = \lambda \tag{32}$$

$$\varsigma' \sim \chi_d^2(\lambda) \tag{33}$$

with d the number of variables in $\mathcal{X}_g$.

While the choice of Mahalonobis norm may be less intuitive than the Euclidean norm in measuring sensitivity, a few comments are in order:

- The original metric does not take into account uncertainty in the individual gradient terms. This metric normalizes each term by its uncertainty, using a sort of "signal-to-noise" measure. This is also appropriate from a Bayesian standpoint, since it downweights uncertain information.

- The original metric does not account for correlation among the gradient terms. However, ς' rotates the original gradient to a basis in which the covariance matrix is diagonal (the PCA basis). Thus, this metric is equivalent to performing a PCA of ∇f and then weighting each component by its inverse variance. (Unlike in PCA, where variance is variance explained, here, variance is uncertainty, so we want to downweight highly variant directions in PCA space.)

- Just as importantly, if $X_1 \sim \chi_{d_1}^2(\lambda_1)$ and $X_2 \sim \chi_{d_2}^2(\lambda_2)$, $X_1 + X_2 \sim \chi_{d_1+d_2}^2(\lambda_1 + \lambda_2)$, so the *average* of a series of (noncentral) χ^2 random variables, $\bar{X}$ is given by

$$N\bar{X} = \sum_i X_i \sim \chi^2_{\sum_i d_i}\left(\sum_i \lambda_i\right) \tag{34}$$

So while $\bar{X}$ *does not* have a conventional distributional form (a rescaled χ^2 is gamma-distributed, but a rescaled noncentral χ^2 is not), its moments and cdf can be obtained by working with $N\bar{X}$ and rescaling appropriately. It is this formula that we use in the main text to calculate credible intervals at each time point when averaging across trials.

Supplementary Note 5: Disentangling identity and context effects in play

When attempting to quantify how player strategy (or indeed, any task-related variable) differs based on the identity of the opponent (human or computer), simply taking the observed difference between the varriable of interest during the human trials and likewise for the computer trials elides an important distinction between what might be termed "opponent identity effects" and "opponent context effects". That is, we might ask whether observed differences in switch probability between the two opponents are due to intrinsic differences in the way participants perceive each opponent or the fact that each opponent simply plays a different strategy. In typical social games, these effects are all but impossible to disentangle, but because we model the joint distribution of both states and opponent identity, $f(s, \omega)$, we can perform the following "counterfactual" experiment: For every state s visited in play against the computer ($\omega = 0$), we can ask how $f(s, 0)$ compares to $f(s, 1)$. This is equivalent to freezing game play at a single moment, switching the identity of the opponent while holding all other variables fixed, and asking how play in the next instant differs. Such a pure identity effect quantifies how much participants' strategies would differ between human and computer opponents who used the same strategy.

More formally, define:

$$\overline{X}_{ab} \equiv \mathbb{E}_{p(\mathbf{s}|\omega=a)}[X(\mathbf{s}, \omega = b)] \tag{35}$$

be an expectation of some random variable X (for instance, a probability of switching or sensitivity). Here again, s represents the game state and ω the opponent identity (0 = computer, 1 = human), but we decouple the opponent specified in the random variable from the opponent that generated the states over which we average. More concretely, $\overline{X}_{00}$ denotes the value of X against the computer, averaged over states actually played against the computer, while $\overline{X}_{10}$ again denotes the value of X against the computer, only this time averaged over states played against the *human*. In this notation, Figure 2D plots $\overline{X}_{00}$ and $\overline{X}_{11}$ with $X = \Phi^{-1}(p)$, while Figure 3 shows the same two variables with X equal to our opponent sensitivity metric.

What is most important, however, is that the observed contrast plotted in purple in Figure 1B-C can be decomposed as a weighted sum of the identity effect C_{identity} and the context effect C_{identity}, as follows:

$$\begin{aligned}
C_{\text{identity}} &\equiv \mathbb{E}_{p(\mathbf{s})}\left[X(\mathbf{s}, 1) - X(\mathbf{s}, 0)\right] \\
&= \frac{n_0}{N}\left(\overline{X}_{01} - \overline{X}_{00}\right) + \frac{n_1}{N}\left(\overline{X}_{11} - \overline{X}_{10}\right) && (36) \\
C_{\text{context}} &\equiv \frac{1}{2}\big[\mathbb{E}_{p(\mathbf{s}|\omega=1)}X(\mathbf{s}, 1) - \mathbb{E}_{p(\mathbf{s}|\omega=0)}X(\mathbf{s}, 1) \\
&\quad + \mathbb{E}_{p(\mathbf{s}|\omega=1)}X(\mathbf{s}, 0) - \mathbb{E}_{p(\mathbf{s}|\omega=0)}X(\mathbf{s}, 0)\big] \\
&= \frac{1}{2}\left(\overline{X}_{11} - \overline{X}_{01}\right) + \frac{1}{2}\left(\overline{X}_{10} - \overline{X}_{00}\right) && (37) \\
C_{\text{observed}} &\equiv \overline{X}_{11} - \overline{X}_{00} \approx C_{\text{identity}} + C_{\text{context}} && (38)
\end{aligned}$$

with n_0 and n_1 the number of trials played against the computer and human opponents, respectively, $N = n_0 + n_1$, and approximate equality holds in Eq 38 because $n_0 \approx n_1$ in our data.

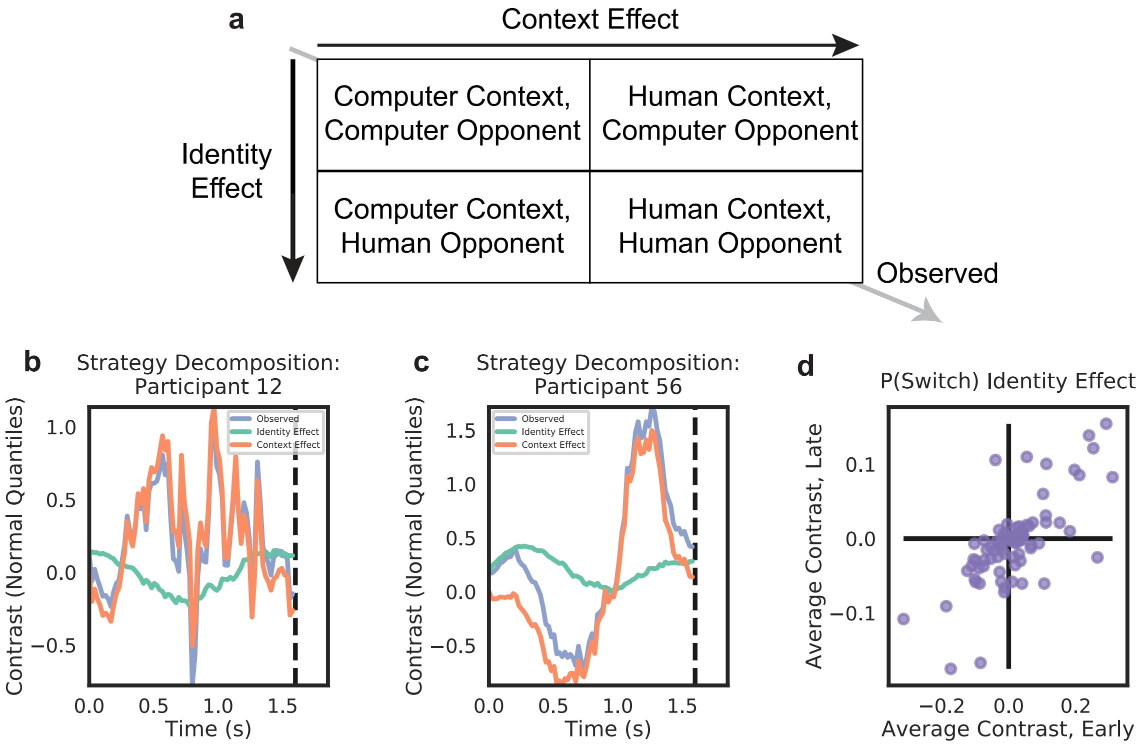

Figure 1: **Disentangling identity and context effects.** A. Schematic of the identity versus context decomposition. Differences in the expected values of model variables between human and computer opponents can be decomposed into a sum of identity and context effects (see Methods). B. Decomposition as a function of time in trial for Participant 12. The difference between human and computer switch probabilities (in quantiles; purple) is the sum of opponent (green) and context (orange) effects. C. Same decomposition as in B, for Participant 56. D. Population variability in opponent effect. Scatter plot of trial-averaged switch probabilities for the first and second half of the trial for each participant. Participants in the upper right consistently switch more against the human opponent, participants in the lower left against the computer. Participants in the other two quadrants switch more frequently against one opponent in the early half of the trial and reverse this behavior in the latter half.

In fact, the observed contrast between the two curves in Figure 2D can be fully decomposed into an effect due to opponent identity and an effect due to differences in the distributions of visited states (see Methods). As indicated in Figure 1A, the observed contrast plotted in Figure 2D corresponds to the difference along the diagonal, while the identity and context effects correspond to differences taken along the vertical and horizontal directions, respectively. Figures 1B and C illustrate this decomposition for two representative participants. These figures show both the observed contrast (difference between the two curves in Figure 2D and its constituent pieces due to opponent identity and context. While the latter are typically larger, indicating a predominance of game state effects on switch probability, there is considerable heterogeneity across both participants and time in trial. Figure 1D illustrates this by considering the average identity effects for each participant during the early and late stages of each trial. There, a positive value indicates higher switch probability for a human opponent, while a negative value indicates higher switch probability against the computer. While some participants consistently exhibit higher switch probabilities

against the human opponent (upper right) or against the computer (lower left), others switch more against one early and the other late (upper left, lower right). Thus, players can be distinguished not only by which opponent elicits more switching behavior, but also by the periods of the trial in which these tendencies occur.

Supplementary Note 6: Empirical Action Value Model

We have shown that we can use nonparametric methods to estimate the policy participants use when playing a dynamic, strategic game. Yet this analysis says nothing about how effective these policies are. So how do participants' choices at each moment translate to wins and losses? To answer this, we separately modeled each participant's action value $Q_\pi(a|\mathbf{s},\omega)$: the expected value of taking action a in state s against opponent ω and playing according to policy π thereafter. As indicated by notation, this value is policy-dependent. That is, each policy π uniquely determines a value function Q_π. In typical reinforcement learning models, policies are likewise dependent on action values: Given action values, Q, policies choose actions based on a softmax function or other rule [4]. Thus, there is a mapping in the reverse direction from action values to policies. The Bellman Equation stipulates that for optimal learners, the optimal policy and action values determine one another [4], but this need not hold for nonoptimal learners.

Figure 2A illustrates these concepts. While the optimal policy π_* and Q_* are mapped onto each other by the processes of value calculation and action selection, respectively, for non-optimal learners, the observed policy π_{obs} leads to a value function Q_{obs}, but softmax action selection based on Q_{obs} may not be equivalent to the original policy: $\pi_Q \neq \pi_{obs}$, so the mappings in Figure 2A are not inverses except for optimal policies. In other words, learners may not necessarily be choosing based on the expected values of their actions. As a result, we took an approach in which the action value function $Q(a|\mathbf{s},\omega)$ was modeled *independently* of π: This model took as inputs the instantaneous state, opponent, and observed action at that time and attempted to predict from those data whether the participant subsequently won the trial. We used the same Gaussian Process classification approach as before, only this time predicting the trial outcome and using the participant's observed action as an additional input.

The results of this model are shown in Figure 2. As Figure 2B illustrates, there are fluctuations in expected value even within a single trial as players move and counter move. Here, we have plotted the predicted expected value, which is equal to the value function of reinforcement learning: $V_\pi(\mathbf{s},\omega) = \sum_a \pi(a|\mathbf{s},\omega)Q_\pi(a|\mathbf{s},\omega)$, an undiscounted, weighted sum of action values according to their probability under the current policy. Quantifying expected value at the time point level, rather than the trial level, allows us to see how fluctuations in game state impact likelihood of winning. For reference, we fit a logistic regression using the same set of input features and targets. Once again, the GP model outperforms logistic regression for each participant in our cohort (see Supplementary Figure 10).

Examining the lengthscale hyperparameter for each input variable answers the questions of whether a given variable is relevant in predicting the target variable in gaussian processes. Large lengthscale values mean a given variable is irrelevant, while smaller values up to approximately 1 mean a variable is predictively relevant. We found that within the empirical expected value GP, 68 out of 82 subjects had an opponent identity lengthscale hyperparameter less than or equal to 1, suggesting that opponent identity impacted expected value for most of the subjects in our task.

Figures 2C and D show these predictions across all trials for a pair of representative participants. Interestingly, while the types of trajectories generated by Participant 3 in both the human and computer opponent conditions look remarkably similar, expected values for these collections of

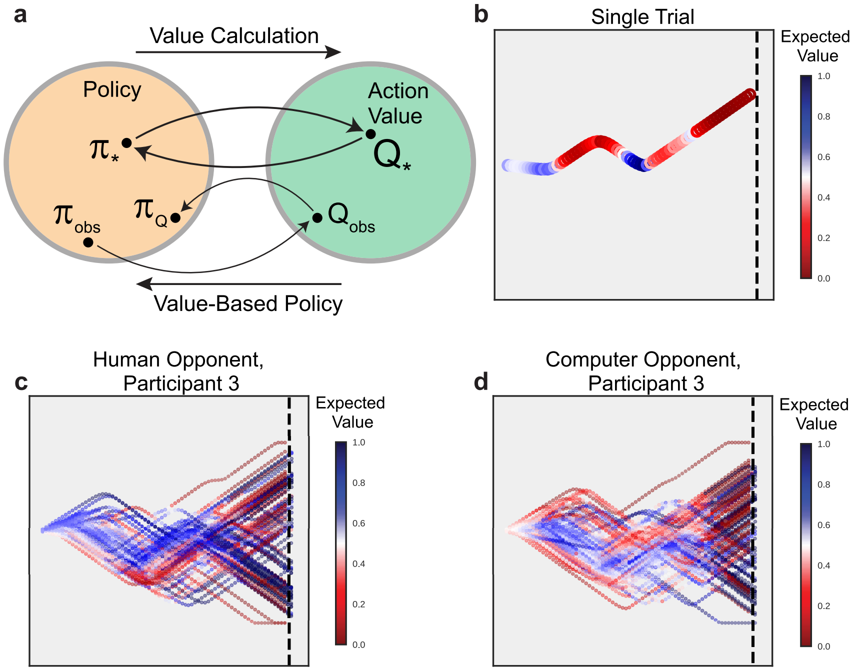

Figure 2: **A Gaussian Process action value model captures variability in player efficacy.** A: Relationship between policies and action values in reinforcement learning. Each policy determines an action value (rightward arrow). Conversely, a set of action values, coupled with an action selection mechanism like softmax or greedy methods, determines a policy (leftward arrow). For optimal learners, the connection between the optimal policy π_* and its resulting action values Q_* is given by the Bellman Equation, which states that the leftward and rightward arrows are inverses of one another. For non-optimal agents, however, the observed policy π_{obs} determines Q_{obs}, but action selection based on Q_{obs} may not be the same as π_{obs}. B: Expected values (win probabilities) at each moment for a single trajectory from one participant. Horizontal and vertical axes correspond to position on the computer screen. Color indicates expected value. C: All trajectories for a single participant against the human opponent. D: Trials against the computer opponent for the same participant as in C. Note the increased intensity of colors late in the trial, after the opponent has made its last move.

trials evolve quite differently. Evidently this participant, while playing essentially the same strategy in both cases, experienced much different win rates against the two opponents. In particular, against the computer opponent, we see a more abrupt transition in expected value between the first and second half of the trial. This can be explained as a byproduct of the computer's "track-then-guess" heuristic, in which it attempts to follow the player in the early and middle stages of the trial and then randomly guesses a direction to move late in play. As a result, during the early and middle phases of the trial, the puck and bar are closely aligned horizontally and expected values hover near 50%. Later, after a "point of no return" at which the computer makes its last decision, expected values are bimodal and concentrated around 0 and 1, reflecting a nearly deterministic outcome.

In fact, this trend can also be visualized in terms of the density of value as a function of time in trial (Figure 3). Against the human opponent (Figure 3A), values start out concentrated around a player's mean win rate and evolve gradually over the course of the trial toward the 0 and 1 outcomes. By contrast, against the computer, values hold around 0.5 until abruptly diverging at the critical

point. And indeed, this pattern holds in the average across all participants (Figure 3C,D). Note that here, in the case of a computer opponent defined by a simple heuristic, our model is easily able to recover strong indications of that heuristic in an unbiased way. This indicates that our approach is powerful enough to characterize a wide range of behavior. Circumstantially, it also suggests that our participants are unlikely to have relied on simple heuristics alone to constructing their strategies.

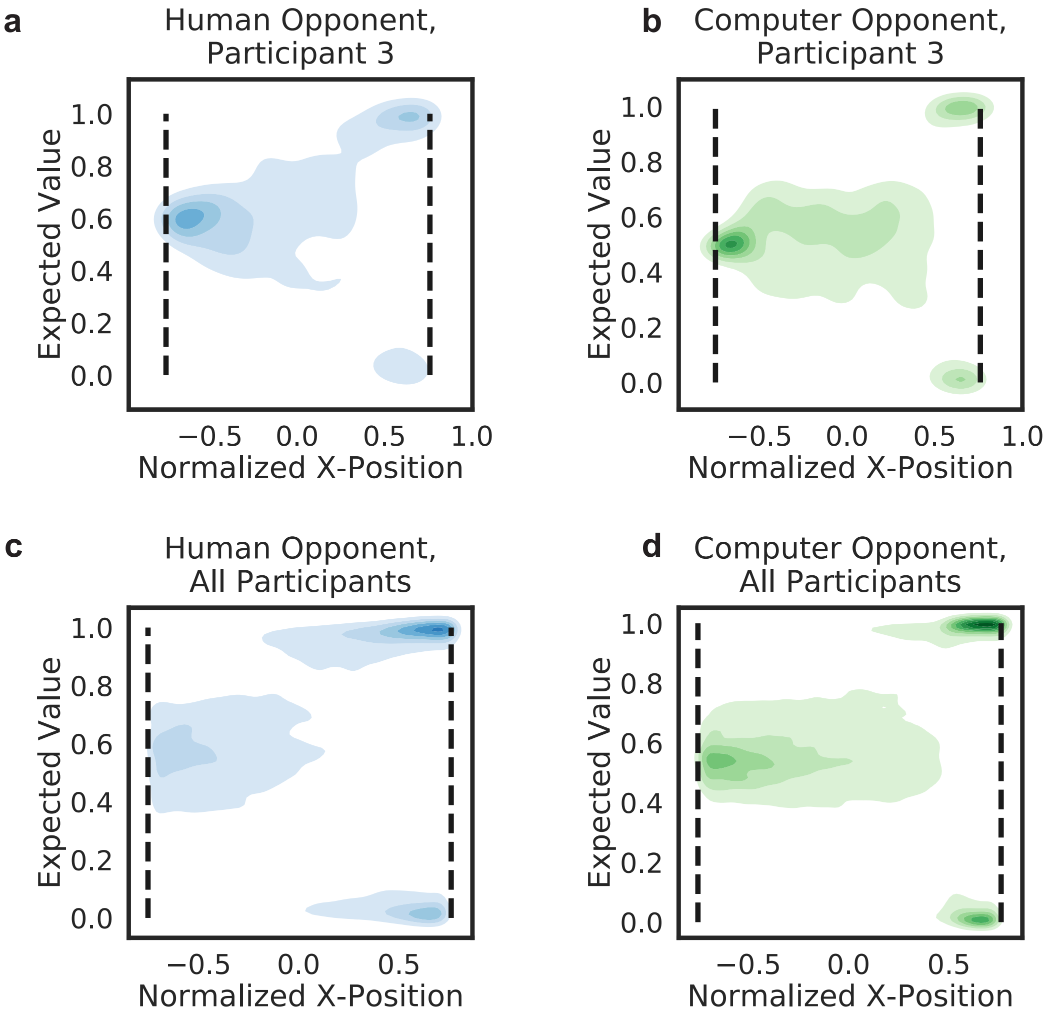

Figure 3: **Evolution of expected value as a function of time in trial.** A, B: Density of expected value as a function of time in trial for a single participant. Color indicates opponent (blue: human, green: computer). Early in the trial, value is concentrated around the participant's baseline win rate for each separate opponent. Over the course of the trial, values grow increasingly bimodal as the participant's prospects for winning diverge based on game state. C, D: Average across all participants. Labeling conventions are as in A, B. Here, the "track-then-guess" heuristic of the computer opponent is apparent in the abrupt transition from a 50% unimodal distribution to a polarized bimodal distribution at the time of the opponent's last move. This bifurcation occurs in trials against the human goalies as well, but earlier than those against the computer goalie.

Finally, to investigate how well expected value predicts whether a given trial will result in a win or loss, we conducted a series of univariate logistic regressions. Given an opponent and an

average expected value in the early, middle, or late periods of each trial, we attempted to predict the trial's result. We found that regression coefficients for the human opponent condition were higher than those for the computer opponent ($t = 4.53$, $p < 0.0001$), suggesting that (unrealized) expected values better predict trial outcome in the human opponent condition. Second, we found that regression coefficients increase as the trial progresses, such that the late coefficients were significantly higher than early coefficients ($t = 30.69$, $p < 0.0001$). This matches our intuition that trial outcomes are better predicted by expected values later in the trial (see Supplementary Figure 11).

Supplementary Note 7: Regularized Logistic Regression

We compared the predictive performance of our classification model to a regularized logistic regression using LASSO as implemented in the scikit-learn `LogisticRegression` class. We fit models with regularization parameters C using 20 evenly spaced values from 10^{-3} to 10^{2} on a $\log_{10}$ scale. For each subject, we selected the model with the highest log likelihood on a test set consisting of 20% held out data.

Supplementary Figures 4-19: Comparison across participants

Here, we reproduce our analyses from the main text for all participants used in any of the figures.

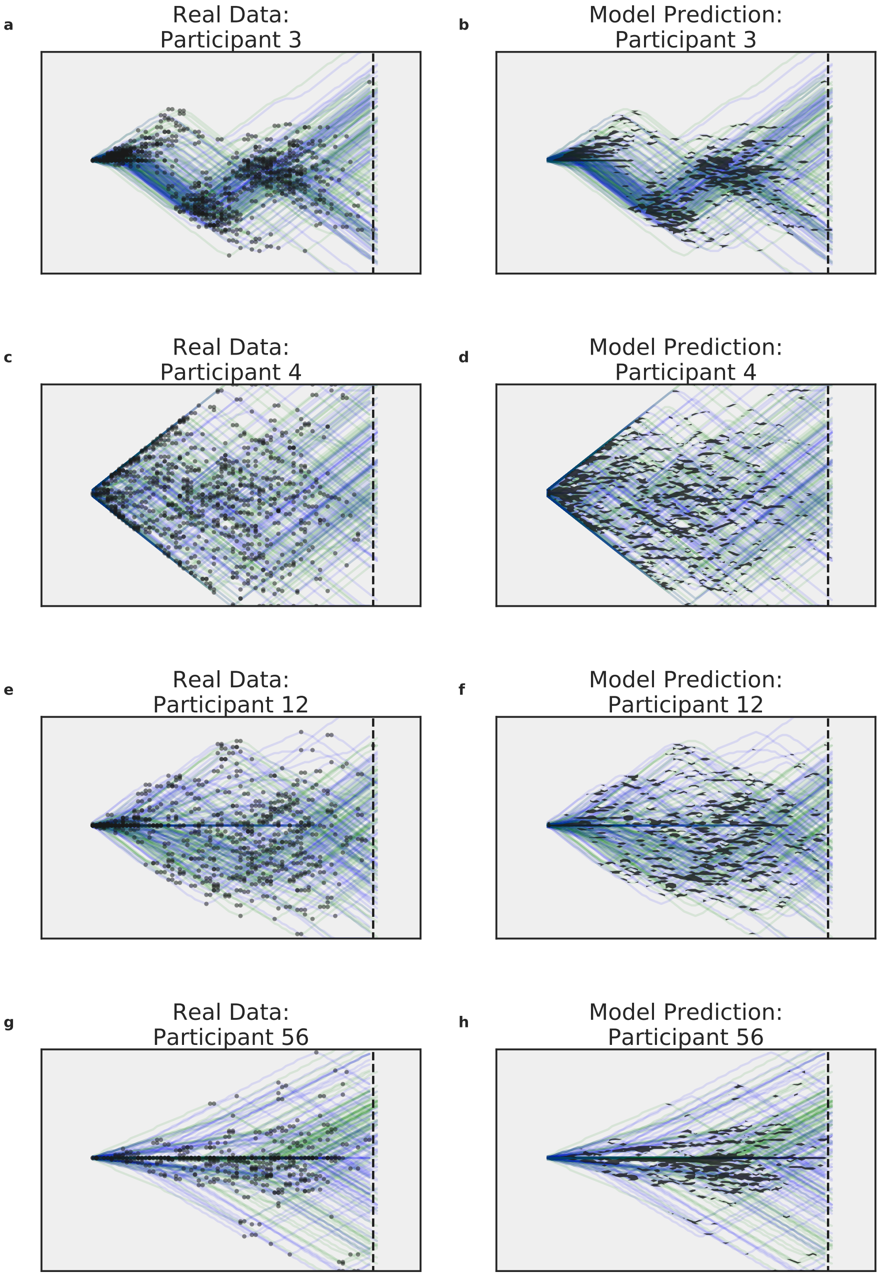

Figure 4: Left column (A,C,E,G): Observed data from the four participants used in figures in the main text. Blue trajectories correspond to trials played against the human opponent. Green trajectories are from trials played against the computer opponent. Black dots represent change points, or switches in joystick direction by the participant. Right column (B,D,F,H): Trajectory data overlaid with black shaded regions indicating increased probability of changepoints in the GP model (greater than the participant's base rate) for the selected participants displayed in the main text.

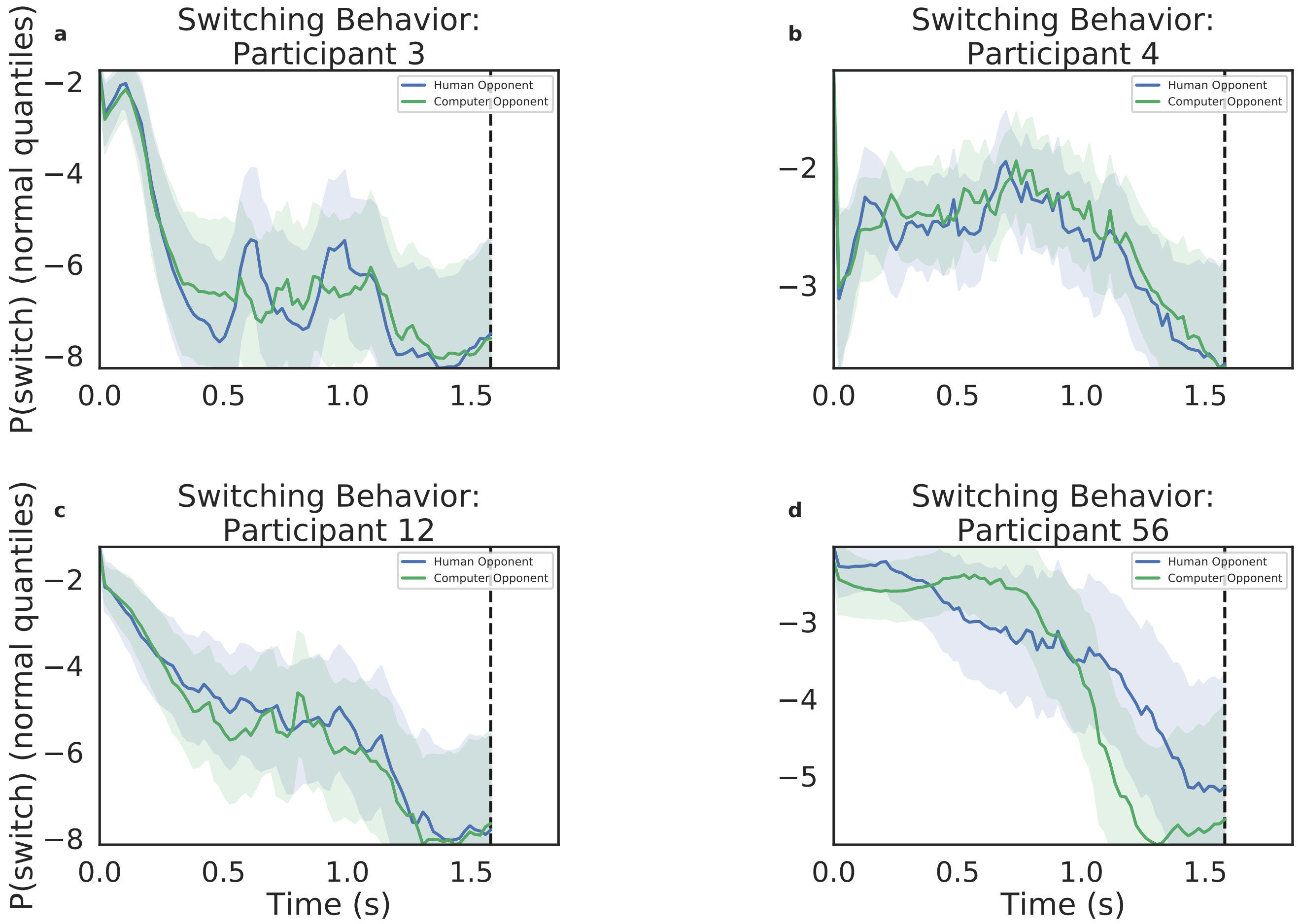

Figure 5: Probability of a change point as a function of time, averaged across trials, for the four selected participants (A,B,C,D) featured in figures in the main text. Shaded regions indicate 95% credible intervals. Probabilities are shown and averaged in quantiles (z) of the normal distribution. Blue indicates trials against the human opponent, green against the computer.

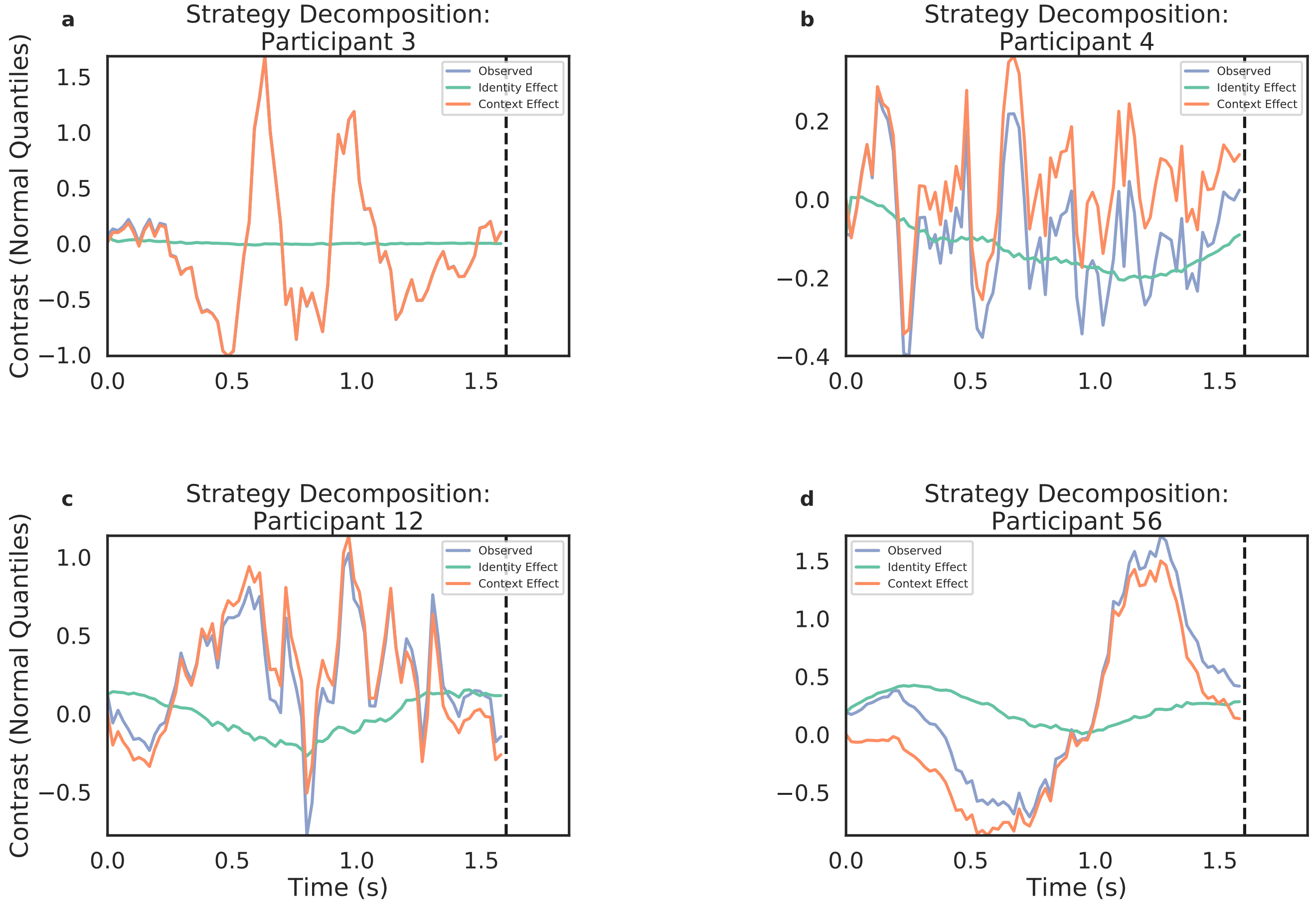

Figure 6: Decomposition of strategy as a function of time in trial for the selected four participants (A,B,C,D). The difference between human and computer switch probabilities (in quantiles; purple) is the sum of opponent (green) and context (orange) effects.

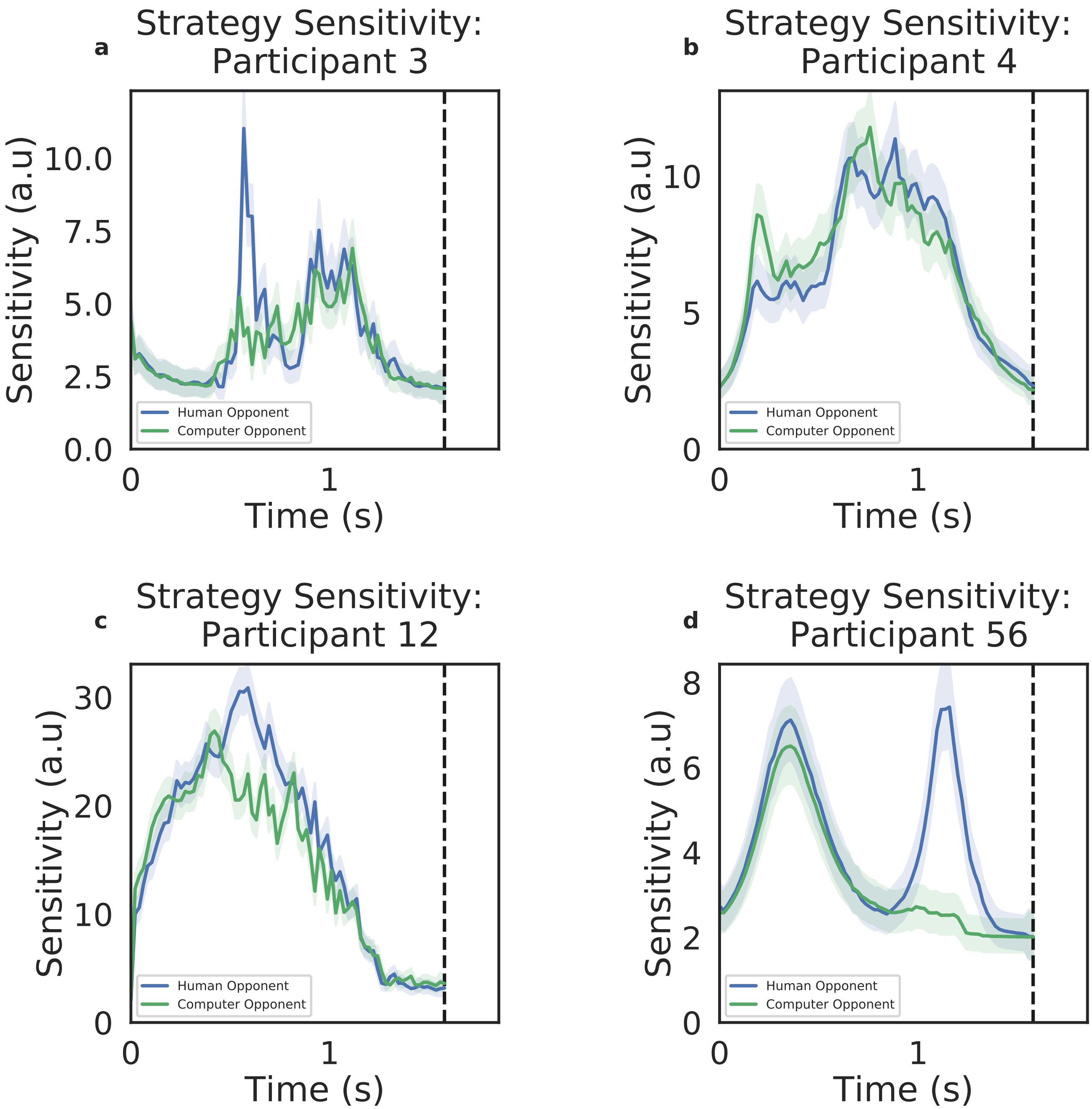

Figure 7: Observed sensitivity to opponent actions in both opponent conditions, for the selected four participants (A,B,C,D). Shaded regions indicate 95% credible intervals. Blue line and shaded region correspond to the human opponent condition, green to the computer opponent.

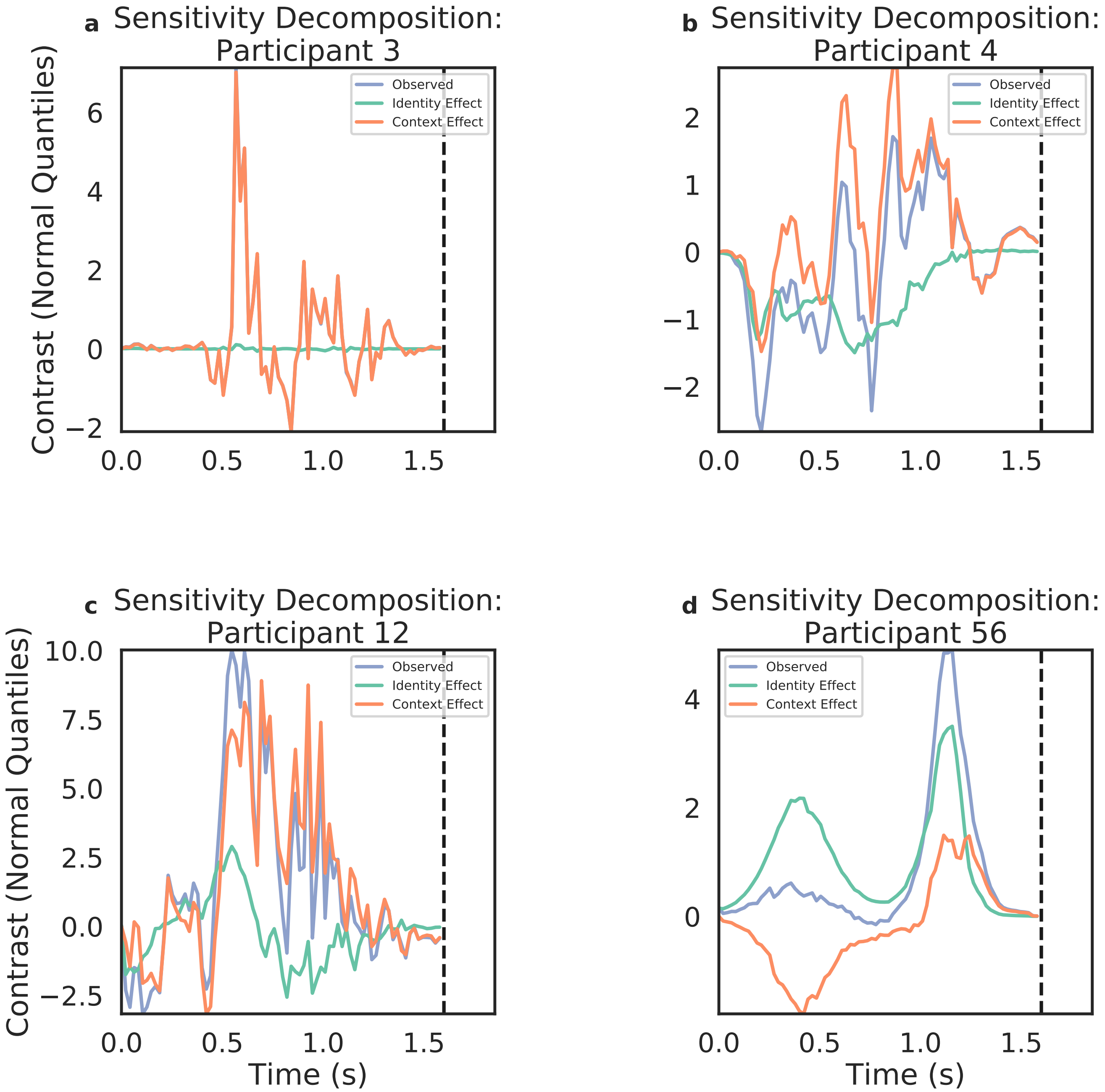

Figure 8: Decomposition of sensitivity to opponent action, with the observed contrast decomposed into identity and context effects for the selected four participants (A,B,C,D).

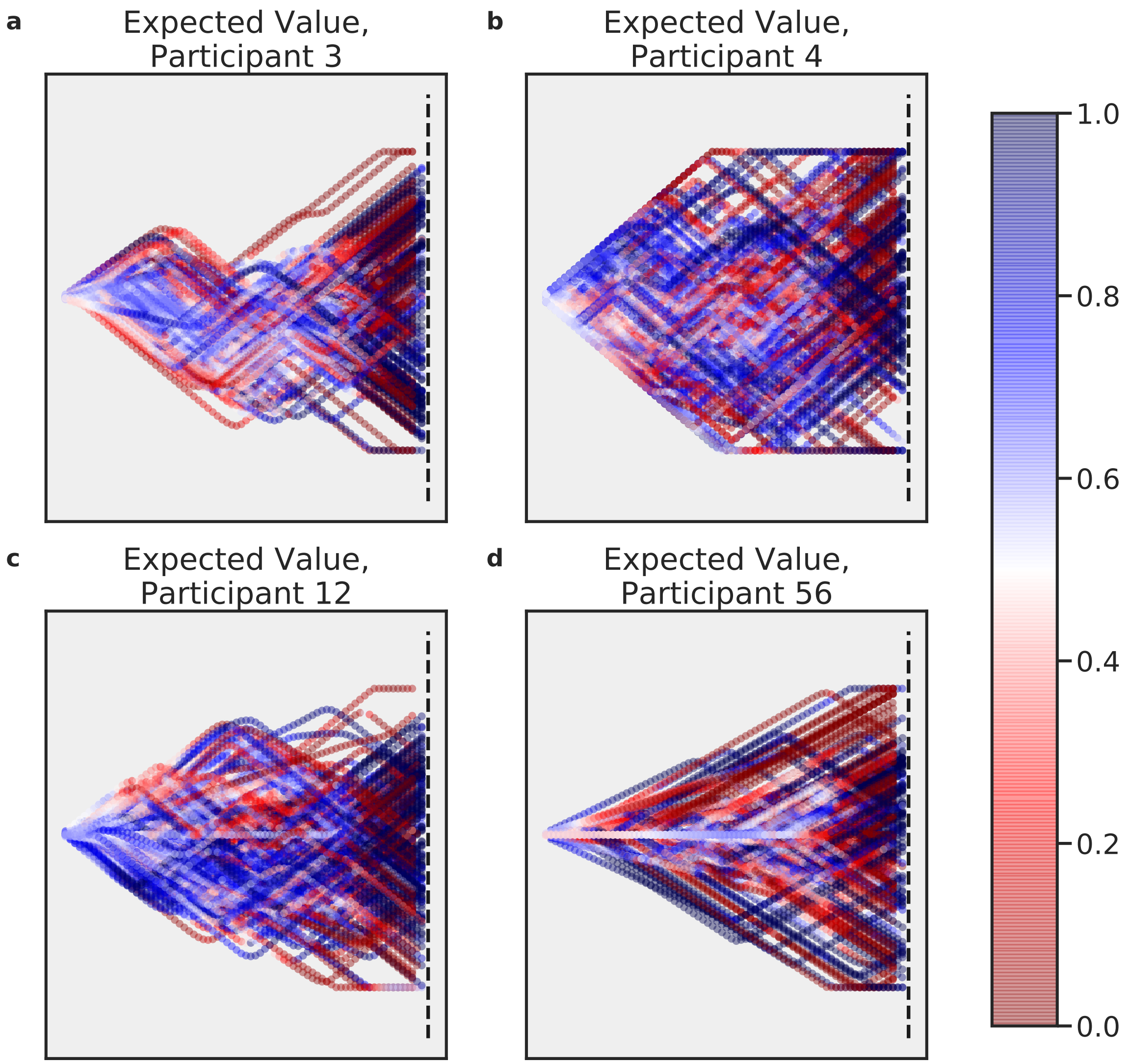

Figure 9: Expected values (win probabilities) at each moment for all trajectories for the featured four participants (A,B,C,D). Horizontal and vertical axes correspond to position on the computer screen. Color indicates expected value.

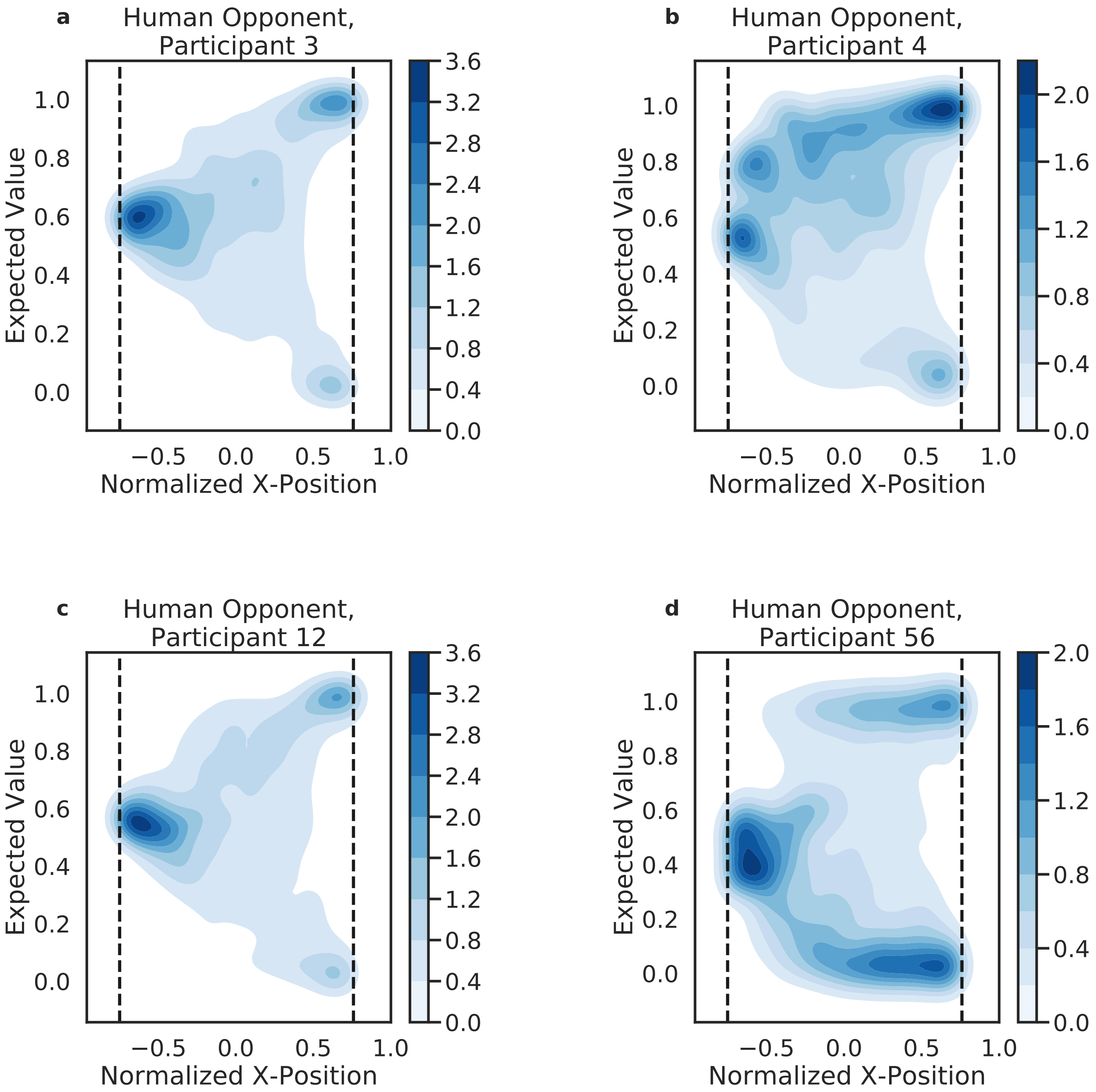

Figure 10: Density of expected value as a function of time in trial for the four featured participants' trials (A,B,C,D) against the human opponent only. Colorbar indicates the density levels, which are determined individually for display purposes.

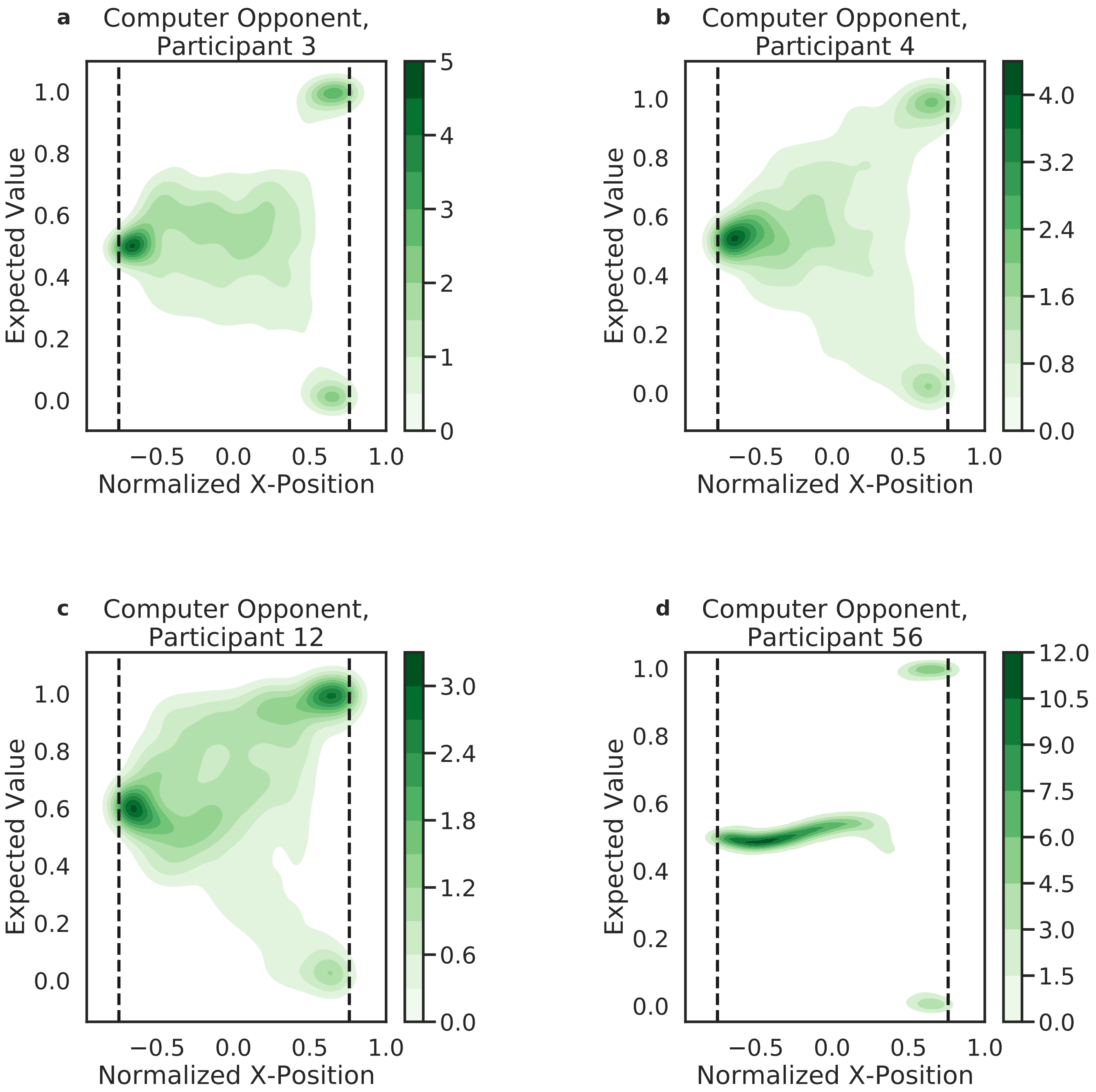

Figure 11: Density of expected value as a function of time in trial for the four featured participants' trials (A,B,C,D) against the computer goalie only. Colorbar indicates the density levels, which are determined individually for display purposes.

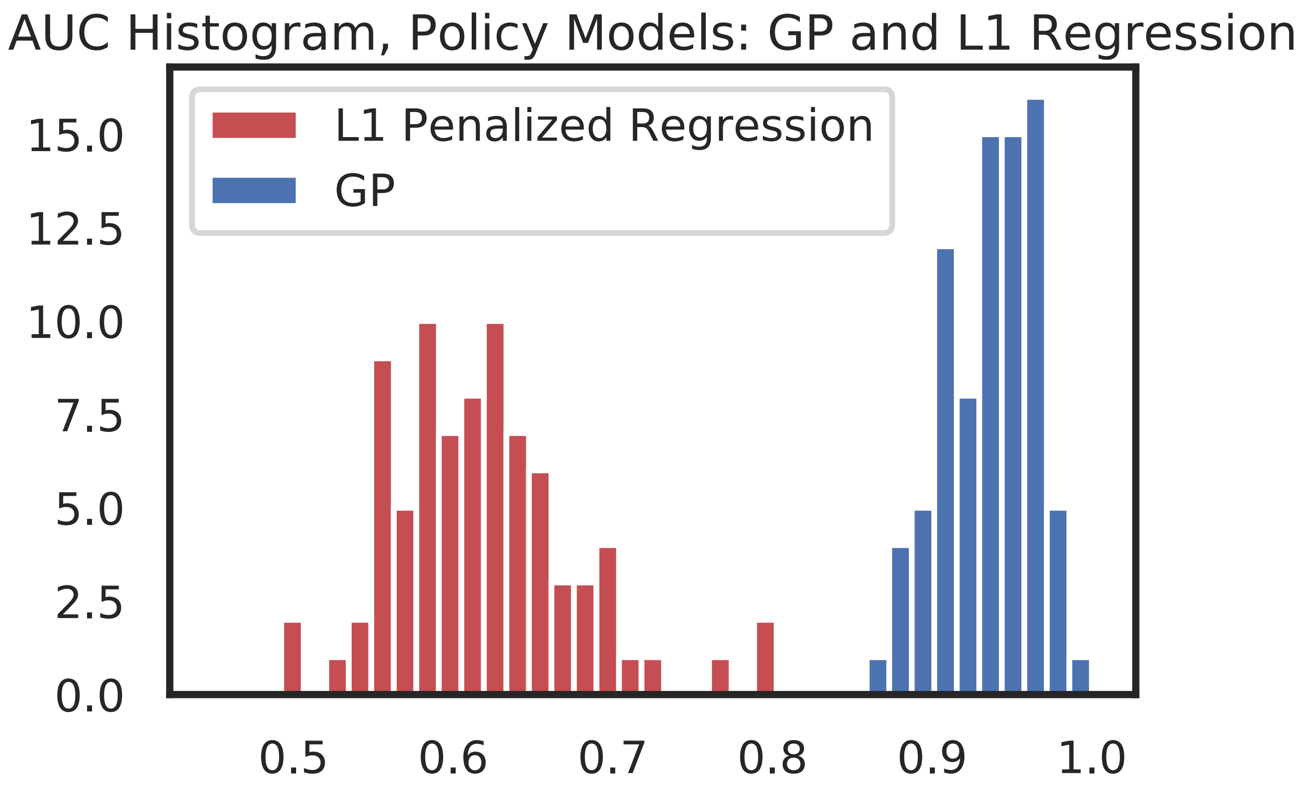

Figure 12: Area under the curve (AUC) for each particpant's Gaussian Process policy model and the corresponding participant's L_1-regularized logistic regression. Every participant's Gaussian Process AUC was higher than that of his/her logistic regression AUC. This is encouraging, though not particularly surprising, since a gaussian process is a non-parametric model and a logistic regression is a linear parametric model.

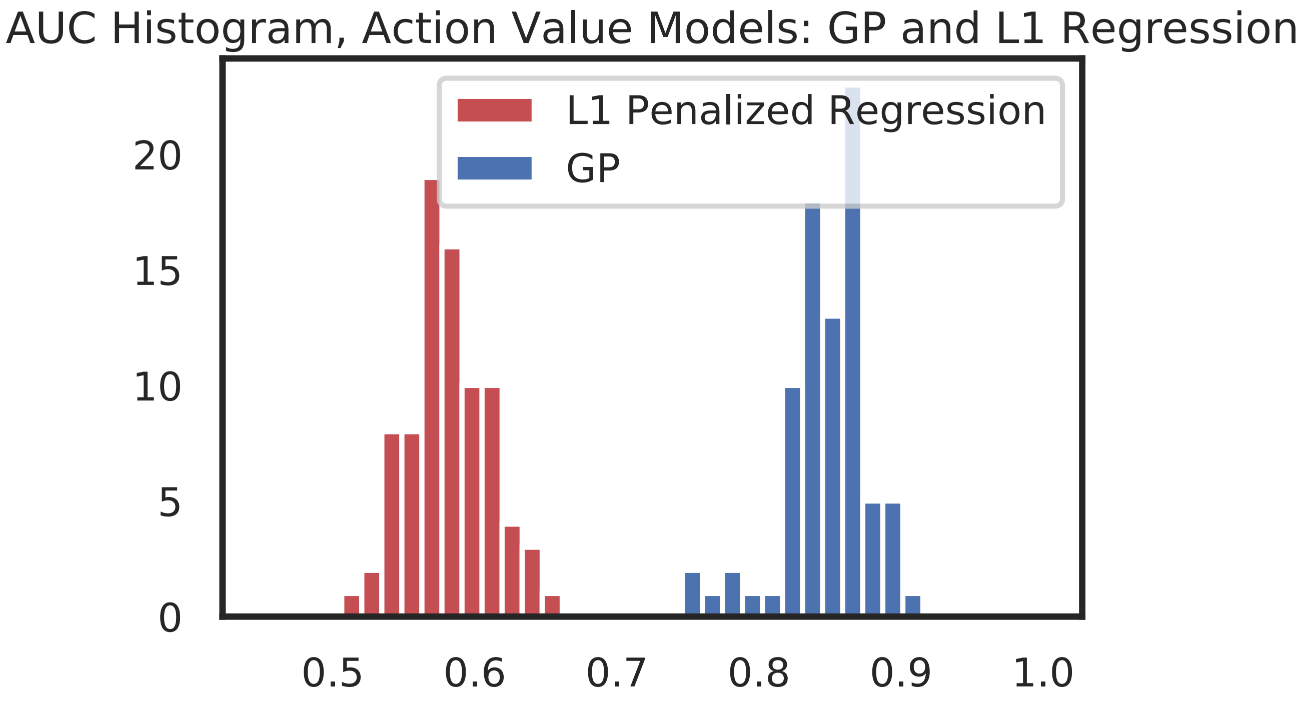

Figure 13: Area under the curve (AUC) for each particpant's empirical value function model and the corresponding participant's L_1-regularized logistic regression. Every participant's Gaussian Process AUC was higher than that of his/her logistic regression AUC.

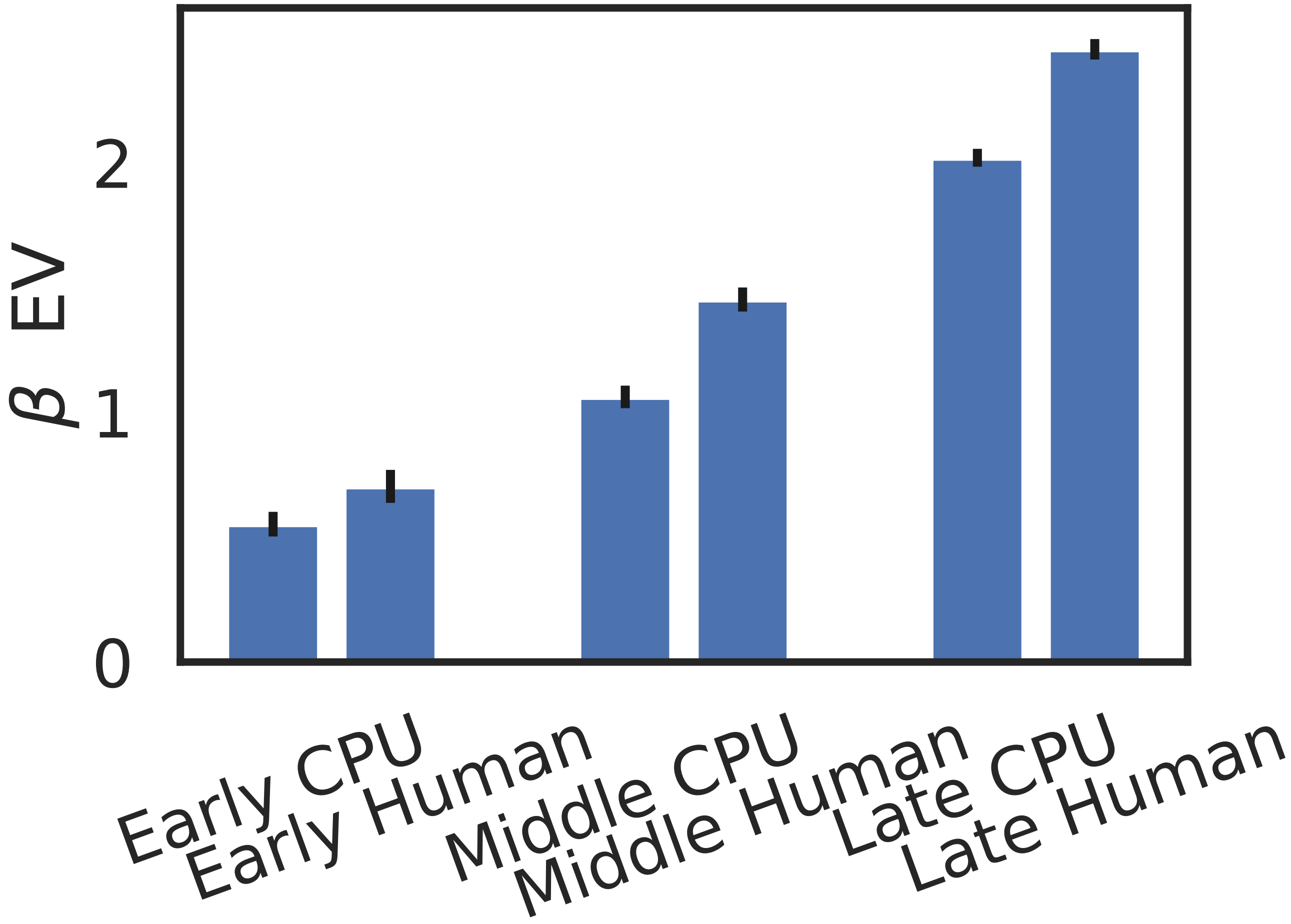

Figure 14: Bar plot of the mean regression coefficient, β for six different regressions. Each univariate regression had the expected value predictions from the action value GP (EV) from certain time periods in certain trials predicting the outcome of the trial. "Early" corresponded to the first third of timepoints in active gameplay, "Middle" is the second third of timepoints, and "Late" is the last third of activate gameplay. Separate regressions were conducted for trials played against the human and computer opponent, and for the separate tertiles of the game. We see that later periods of game play have a higher, positive coefficient predicting the outcome of the trial, and that overall, coefficients are higher for the human trials than the computer trials. Error bars, s.e.m.

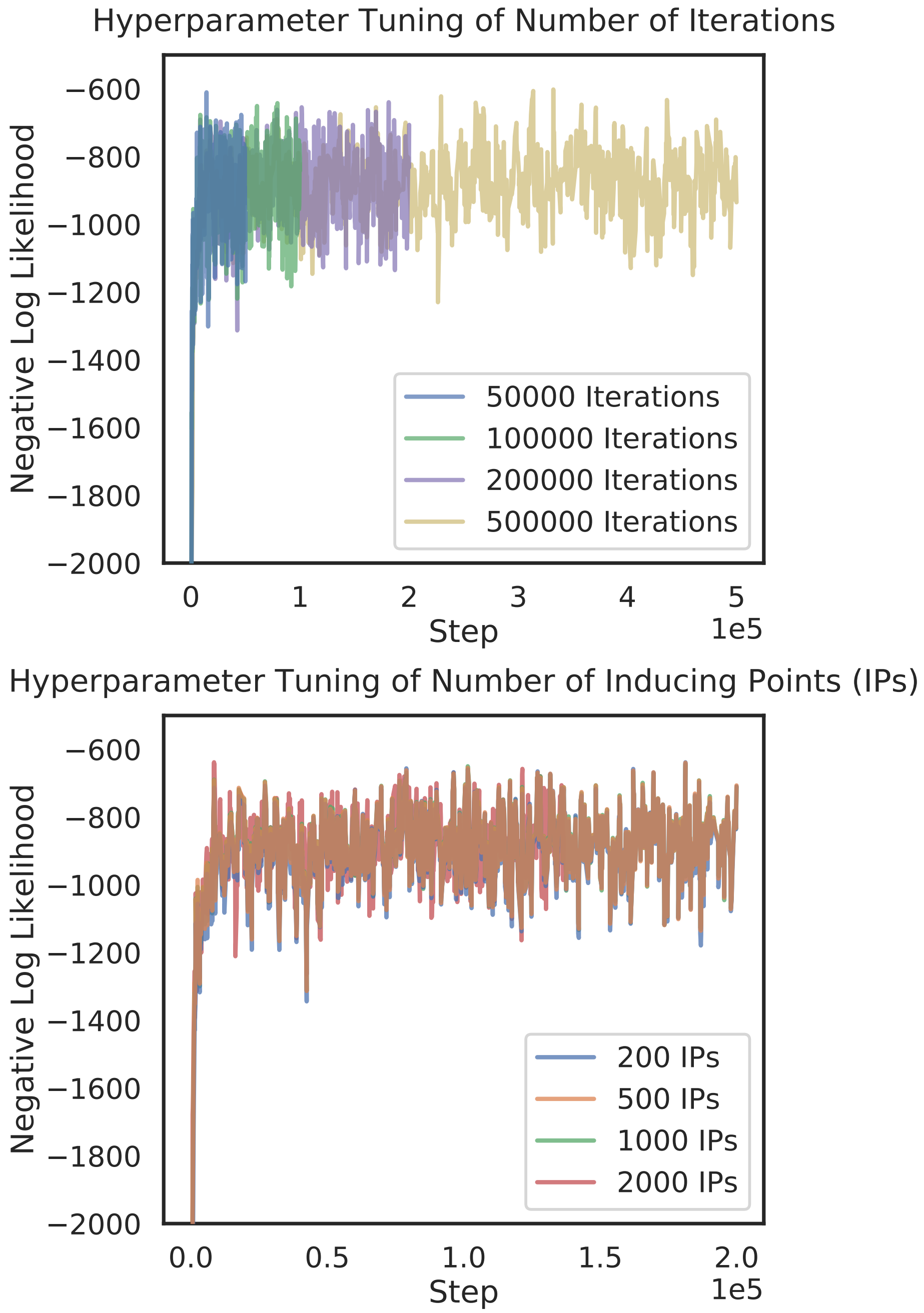

Figure 15: Top: Learning curves from the same participant's data, with number of inducing points and random seed held constant. The only variable changed in the top plot is the number of iterations used to train the GP. We ultimately used 200,000 iterations to train each participant's GP, since 500,000 iterations did not improve the log likelihood of training. Bottom: Learning curves from the same participant'ss data, with the number of inducing points (IPs) being the only variable that is changed. 500 IPs were used for the final models, since adding inducing points past this point did not significantly increase log likelihood.

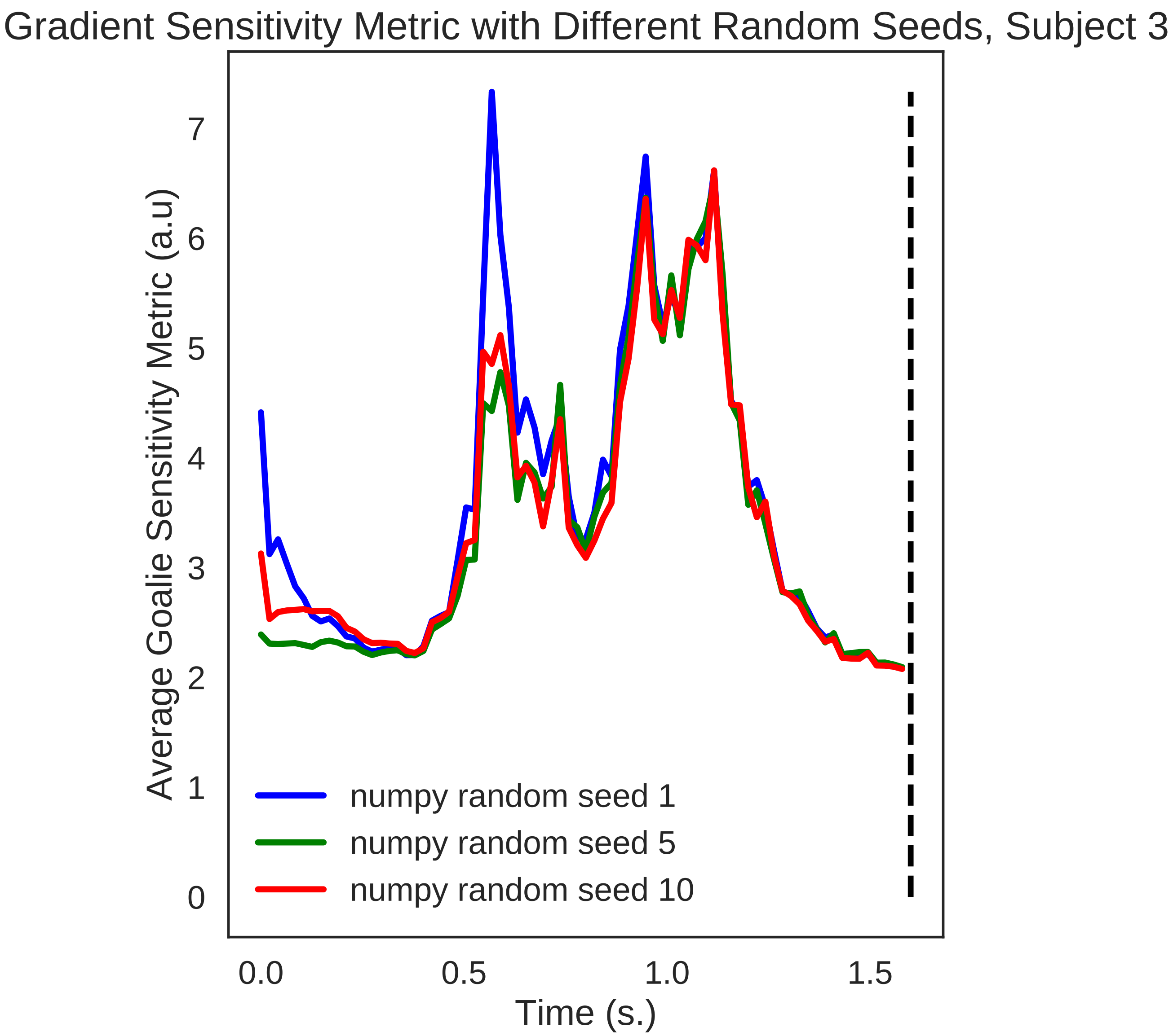

Figure 16: Gaussian Process policy model gradients robust to different random seeds. One participant's behavioral data was fit with three separate Gaussian Process models with all hyper-parameters held constant (participant 3, 500 inducing points, 200,000 iterations, 256 minibatch size), except 3 different random seeds were used (with the numpy package). This was conducted to determine how sensitive the gradient sensitivity to opponent actions metric was to random seed changes. We find that the qualitative form of the gradient shape remains the same and the conclusions drawn regarding the timing and amplitude of the gradient sensitivity curve are not impacted significantly by changes in random seeds used.

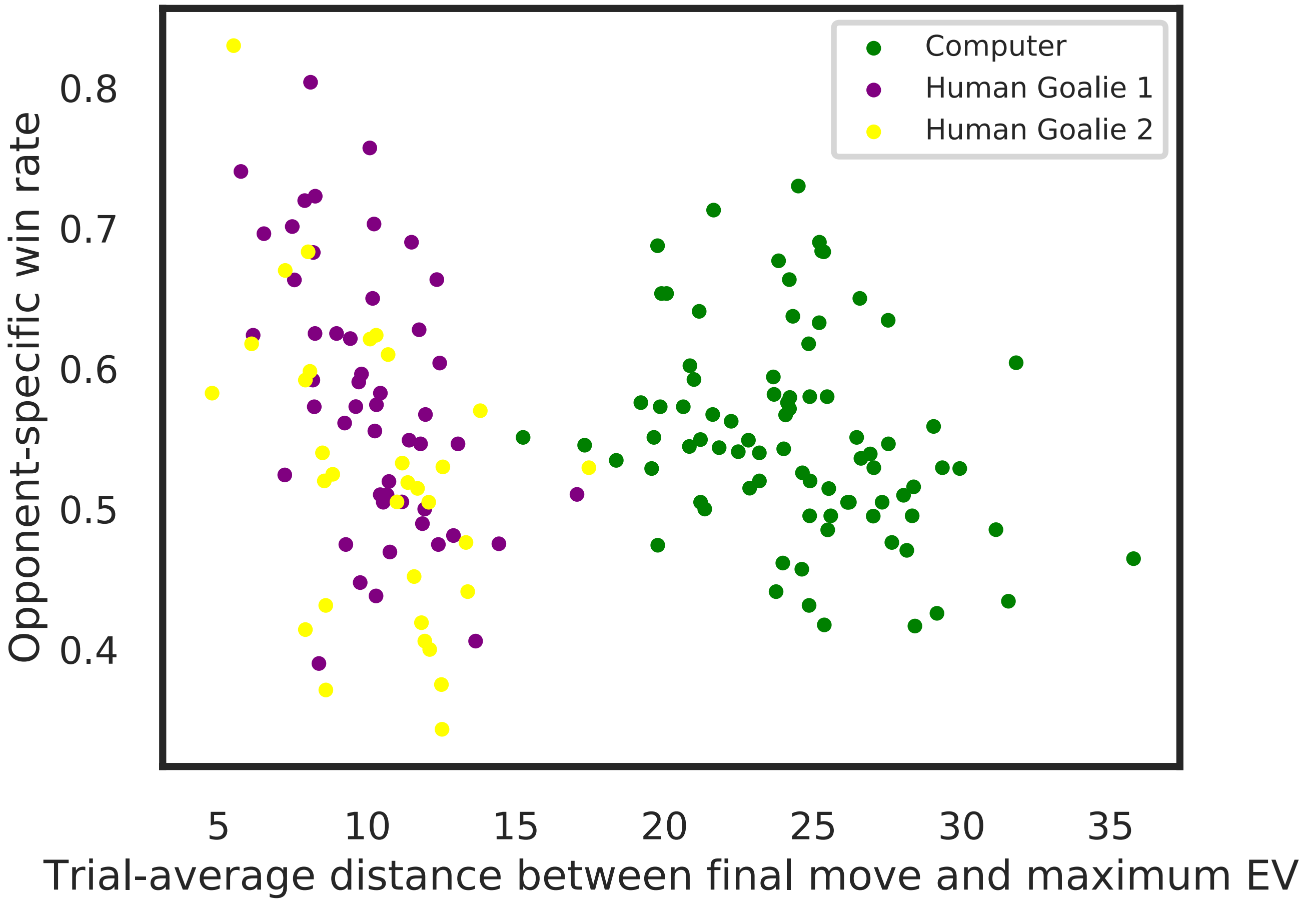

Figure 17: Scatter plot of subject-average distance in time between each trial's final change in direction and that trial's time index in which the expected value of making a final move is at its maximum. Each subject in our dataset is represented as two dots: that subject's distance average against the computer goalie and that particular subject's human goalie (either human goalie 1 or human goalie 2).

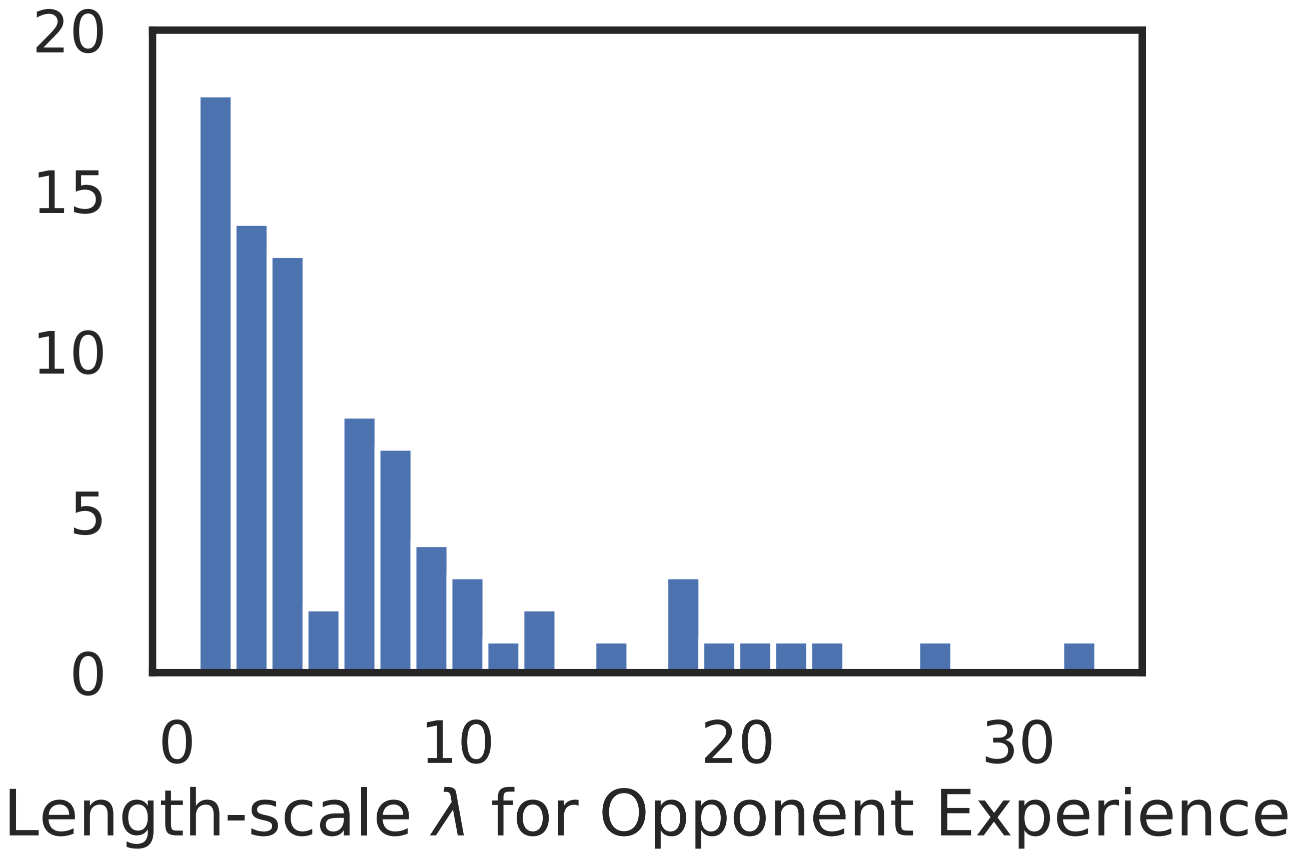

Figure 18: Histogram of each subject's Opponent Experience length-scale hyperparameter from his/her Policy Gaussian Process model. Large values mean that particular subject's likelihood of switching directions was relatively unaffected by how much experience that subject had playing against either the human or computer goalie.

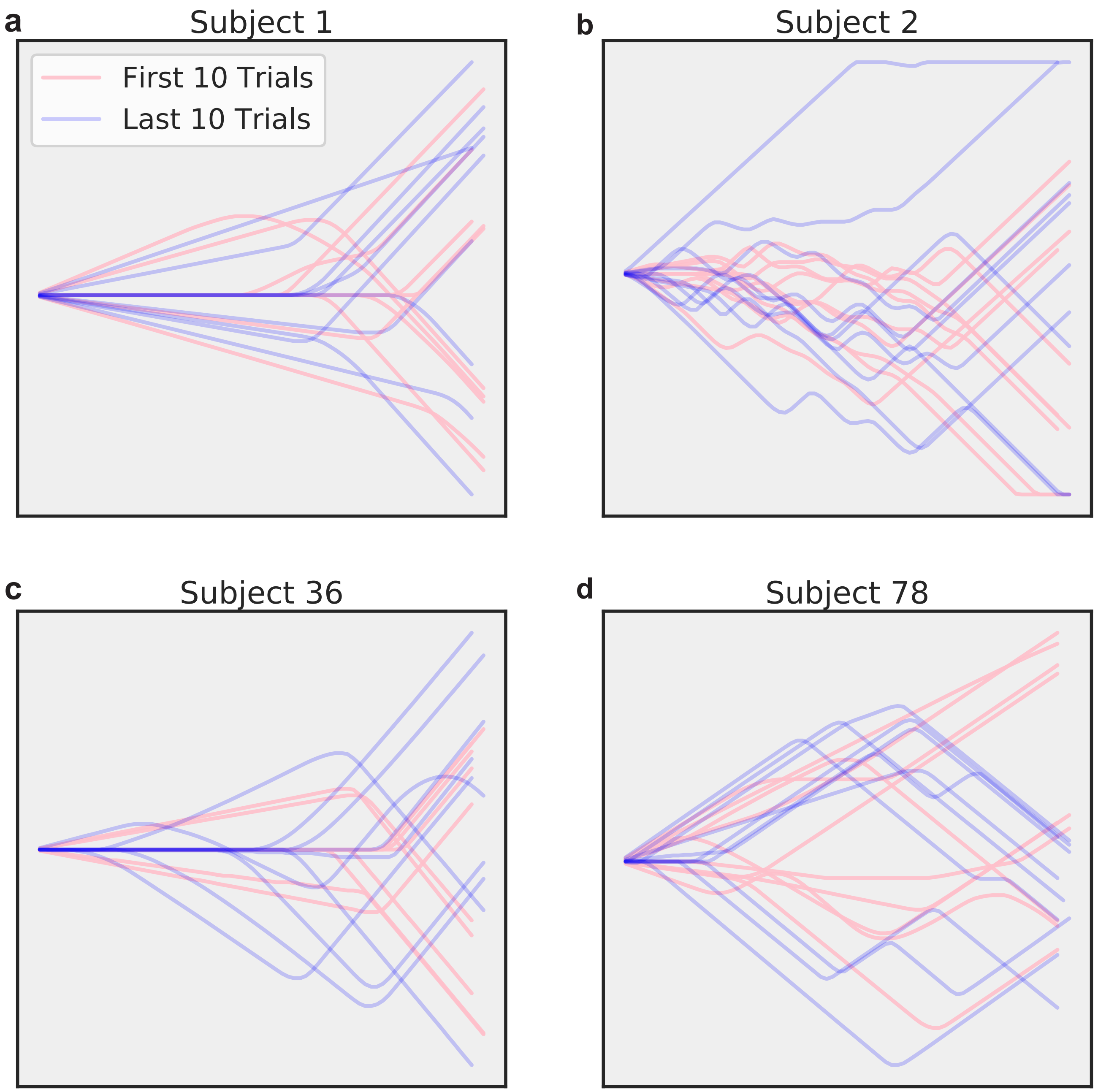

Figure 19: Descriptive plots of four subjects' (A,B,C,D) first 10 trial trajectories (colored in pink) and last 10 trial trajectories (colored in blue). Early- and late-session trials show very little descriptive difference in terms of spatial position.

References

[1] Iqbal, S. & Pearson, J. A goal-based movement model for continuous multi-agent tasks. Preprint at *arXiv:1702.07319* (2017).

[2] Rasmussen, C. E. & Williams, C. K. *Gaussian process for machine learning* (MIT press, 2006).

[3] Baldessari, B. The distribution of a quadratic form of normal random variables. *The Annals of Mathematical Statistics* **38**, 1700–1704 (1967).

[4] Sutton, R. S. & Barto, A. G. *Reinforcement learning: An introduction* (MIT press Cambridge, 1998).
