# Supplementary material for: Bayesian nonparametric models characterize instantaneous strategies in a competitive dynamic game
Source: Nat Commun. 2019 Apr 18;10:1808. doi: 10.1038/s41467-019-09789-4 (PMC6472387; doi:10.1038/s41467-019-09789-4)
Supplement: Supplementary file 3 — Reporting Summary [file 41467_2019_9789_MOESM3_ESM.pdf]

nature research

Corresponding author(s): DBPR; NCOMMS-18-23344B

Last updated by author(s): 03/16/2019

Reporting Summary

Nature Research wishes to improve the reproducibility of the work that we publish. This form provides structure for consistency and transparency in reporting. For further information on Nature Research policies, see Authors & Referees and the Editorial Policy Checklist.

Statistics

For all statistical analyses, confirm that the following items are present in the figure legend, table legend, main text, or Methods section.

n/a	Confirmed	
☐	☒	The exact sample size (*n*) for each experimental group/condition, given as a discrete number and unit of measurement
☐	☒	A statement on whether measurements were taken from distinct samples or whether the same sample was measured repeatedly
☐	☒	The statistical test(s) used AND whether they are one- or two-sided<br>*Only common tests should be described solely by name; describe more complex techniques in the Methods section.*
☐	☒	A description of all covariates tested
☐	☒	A description of any assumptions or corrections, such as tests of normality and adjustment for multiple comparisons
☐	☒	A full description of the statistical parameters including central tendency (e.g. means) or other basic estimates (e.g. regression coefficient) AND variation (e.g. standard deviation) or associated estimates of uncertainty (e.g. confidence intervals)
☐	☒	For null hypothesis testing, the test statistic (e.g. *F*, *t*, *r*) with confidence intervals, effect sizes, degrees of freedom and *P* value noted<br>*Give P values as exact values whenever suitable.*
☐	☒	For Bayesian analysis, information on the choice of priors and Markov chain Monte Carlo settings
☐	☒	For hierarchical and complex designs, identification of the appropriate level for tests and full reporting of outcomes
☐	☒	Estimates of effect sizes (e.g. Cohen's *d*, Pearson's *r*), indicating how they were calculated

Our web collection on statistics for biologists contains articles on many of the points above.

Software and code

Policy information about availability of computer code

Data collection	Data was collected in an fMRI scanner. Task code was developed in Matlab.
Data analysis	Analysis was performed using custom scripts created using GPflow and Python. Software is publicly available on Github (https://github.com/krm58/PenaltyShot_Behavior), and data are available on Open Science Framework (DOI: 10.17605/OSF.IO/EVFG5).

For manuscripts utilizing custom algorithms or software that are central to the research but not yet described in published literature, software must be made available to editors/reviewers. We strongly encourage code deposition in a community repository (e.g. GitHub). See the Nature Research guidelines for submitting code & software for further information.

Data

Policy information about availability of data

All manuscripts must include a data availability statement. This statement should provide the following information, where applicable:

- Accession codes, unique identifiers, or web links for publicly available datasets
- A list of figures that have associated raw data
- A description of any restrictions on data availability

Preprocessed data are available on Open Science Framework (DOI: 10.17605/OSF.IO/EVFG5).

Field-specific reporting

Please select the one below that is the best fit for your research. If you are not sure, read the appropriate sections before making your selection.

☐ Life sciences ☒ Behavioural & social sciences ☐ Ecological, evolutionary & environmental sciences

Behavioural & social sciences study design

All studies must disclose on these points even when the disclosure is negative.

Study description	Quantitative experimental.
Research sample	The research sample was collected from Duke University and the local community. Every participant was required to meet eligibility requirements for fMRI scans. 82 subjects total; age range: 18-48 years; 45 females; 37 males).
Sampling strategy	No sampling procedure for participants was needed: All 82 participants engaged in the same task conditions. No sample-size calculation was performed; sample sizes were chosen at twice the size of similar neuroimaging studies.
Data collection	Data collection was conducted at the Brain Imaging Analysis Center at Duke. Subjects viewed a computer screen inside the scanner and used a joystick to perform the task. An imaging technician was present outside of the scanner while the experiment was conducted. The research was not blind to experimental condition (whether the participant was playing against the human or computer opponent).
Timing	Data were collected in two cohorts: Cohort 1 collected from 07-26-2016 to 02-14-2017; Cohort 2 collected from 10-20-2017 to 04-20-2018.
Data exclusions	No data were excluded from analyses.
Non-participation	No participants declined to participate; potential participants were screened for fMRI eligibility criteria prior to scheduling.
Randomization	Participants were not randomized to groups, since the study was a within-subjects design.

Reporting for specific materials, systems and methods

We require information from authors about some types of materials, experimental systems and methods used in many studies. Here, indicate whether each material, system or method listed is relevant to your study. If you are not sure if a list item applies to your research, read the appropriate section before selecting a response.

Materials & experimental systems

n/a	Involved in the study	
☒	☐	Antibodies
☒	☐	Eukaryotic cell lines
☒	☐	Palaeontology
☒	☐	Animals and other organisms
☐	☒	Human research participants
☒	☐	Clinical data

Methods

n/a	Involved in the study	
☒	☐	ChIP-seq
☒	☐	Flow cytometry
☒	☐	MRI-based neuroimaging

Human research participants

Policy information about studies involving human research participants

Population characteristics	See above.
Recruitment	Participants were recruited by the Brain Imaging Analysis Center at Duke by a third-party scheduling coordinator. Both Duke and non-Duke affiliated participants were collected in order to collect a representative sample.
Ethics oversight	Institutional Review Board of Duke University Medical Center

Note that full information on the approval of the study protocol must also be provided in the manuscript.
