# Supplementary material for: Bayesian nonparametric models characterize instantaneous strategies in a competitive dynamic game
Source: Nat Commun. 2019 Apr 18;10:1808. doi: 10.1038/s41467-019-09789-4 (PMC6472387; doi:10.1038/s41467-019-09789-4)
Supplement: Supplementary file 4 — Description of Additional Supplementary Files [file 41467_2019_9789_MOESM4_ESM.pdf]

Description of Additional Supplementary Files

File Name: Supplementary Movie 1
Description: Compilation of observed trajectories from participants playing the Penalty Shot task. Participants start each trial on the left-hand side of the screen, traveling towards the right-hand side of the screen at a constant horizontal velocity. The goalie has a fixed horizontal position near the right-hand side of the screen in front of the goal line.
